# A search for decays of the Higgs boson to invisible particles in events with a top-antitop quark pair or a vector boson in proton-proton collisions at $\sqrt{s} = 13\,\text {Te}\hspace{-.08em}\text {V}$

**DOI:** 10.1140/epjc/s10052-023-11952-7

**Published:** 2023-10-16

**Authors:** A. Tumasyan, W. Adam, J. W. Andrejkovic, T. Bergauer, S. Chatterjee, K. Damanakis, M. Dragicevic, A. Escalante Del Valle, P. S. Hussain, M. Jeitler, N. Krammer, L. Lechner, D. Liko, I. Mikulec, P. Paulitsch, J. Schieck, R. Schöfbeck, D. Schwarz, M. Sonawane, S. Templ, W. Waltenberger, C.-E. Wulz, M. R. Darwish, T. Janssen, T. Kello, H. Rejeb Sfar, P. Van Mechelen, E. S. Bols, J. D’Hondt, A. De Moor, M. Delcourt, H. El Faham, S. Lowette, A. Morton, D. Müller, A. R. Sahasransu, S. Tavernier, W. Van Doninck, S. Van Putte, D. Vannerom, B. Clerbaux, S. Dansana, G. De Lentdecker, L. Favart, D. Hohov, J. Jaramillo, K. Lee, M. Mahdavikhorrami, I. Makarenko, A. Malara, S. Paredes, L. Pétré, N. Postiau, L. Thomas, M. Vanden Bemden, C. Vander Velde, P. Vanlaer, D. Dobur, J. Knolle, L. Lambrecht, G. Mestdach, C. Rendón, A. Samalan, K. Skovpen, M. Tytgat, N. Van Den Bossche, B. Vermassen, L. Wezenbeek, A. Benecke, G. Bruno, F. Bury, C. Caputo, P. David, C. Delaere, I. S. Donertas, A. Giammanco, K. Jaffel, Sa. Jain, V. Lemaitre, K. Mondal, A. Taliercio, T. T. Tran, P. Vischia, S. Wertz, G. A. Alves, E. Coelho, C. Hensel, A. Moraes, P. Rebello Teles, W. L. Aldá Júnior, M. Alves Gallo Pereira, M. Barroso Ferreira Filho, H. Brandao Malbouisson, W. Carvalho, J. Chinellato, E. M. Da Costa, G. G. Da Silveira, D. De Jesus Damiao, V. Dos Santos Sousa, S. Fonseca De Souza, J. Martins, C. Mora Herrera, K. Mota Amarilo, L. Mundim, H. Nogima, A. Santoro, S. M. Silva Do Amaral, A. Sznajder, M. Thiel, A. Vilela Pereira, C. A. Bernardes, L. Calligaris, T. R. Fernandez Perez Tomei, E. M. Gregores, P. G. Mercadante, S. F. Novaes, Sandra S. Padula, A. Aleksandrov, G. Antchev, R. Hadjiiska, P. Iaydjiev, M. Misheva, M. Rodozov, M. Shopova, G. Sultanov, A. Dimitrov, T. Ivanov, L. Litov, B. Pavlov, P. Petkov, A. Petrov, E. Shumka, S. Thakur, T. Cheng, T. Javaid, M. Mittal, L. Yuan, M. Ahmad, G. Bauer, Z. Hu, S. Lezki, K. Yi, G. M. Chen, H. S. Chen, M. Chen, F. Iemmi, C. H. Jiang, A. Kapoor, H. Liao, Z.-A. Liu, V. Milosevic, F. Monti, R. Sharma, J. Tao, J. Thomas-Wilsker, J. Wang, H. Zhang, J. Zhao, A. Agapitos, Y. An, Y. Ban, A. Levin, C. Li, Q. Li, X. Lyu, Y. Mao, S. J. Qian, X. Sun, D. Wang, J. Xiao, H. Yang, M. Lu, Z. You, N. Lu, X. Gao, D. Leggat, H. Okawa, Y. Zhang, Z. Lin, C. Lu, M. Xiao, C. Avila, D. A. Barbosa Trujillo, A. Cabrera, C. Florez, J. Fraga, J. Mejia Guisao, F. Ramirez, M. Rodriguez, J. D. Ruiz Alvarez, D. Giljanovic, N. Godinovic, D. Lelas, I. Puljak, Z. Antunovic, M. Kovac, T. Sculac, V. Brigljevic, B. K. Chitroda, D. Ferencek, S. Mishra, M. Roguljic, A. Starodumov, T. Susa, A. Attikis, K. Christoforou, S. Konstantinou, J. Mousa, C. Nicolaou, F. Ptochos, P. A. Razis, H. Rykaczewski, H. Saka, A. Stepennov, M. Finger, M. Finger Jr., A. Kveton, E. Ayala, E. Carrera Jarrin, A. A. Abdelalim, E. Salama, M. Abdullah Al-Mashad, M. A. Mahmoud, S. Bhowmik, R. K. Dewanjee, K. Ehataht, M. Kadastik, T. Lange, S. Nandan, C. Nielsen, J. Pata, M. Raidal, L. Tani, C. Veelken, P. Eerola, H. Kirschenmann, K. Osterberg, M. Voutilainen, S. Bharthuar, E. Brücken, F. Garcia, J. Havukainen, M. S. Kim, R. Kinnunen, T. Lampén, K. Lassila-Perini, S. Lehti, T. Lindén, M. Lotti, L. Martikainen, M. Myllymäki, M. M. Rantanen, H. Siikonen, E. Tuominen, J. Tuominiemi, P. Luukka, H. Petrow, T. Tuuva, C. Amendola, M. Besancon, F. Couderc, M. Dejardin, D. Denegri, J. L. Faure, F. Ferri, S. Ganjour, P. Gras, G. Hamel de Monchenault, V. Lohezic, J. Malcles, J. Rander, A. Rosowsky, M.Ö. Sahin, A. Savoy-Navarro, P. Simkina, M. Titov, C. Baldenegro Barrera, F. Beaudette, A. Buchot Perraguin, P. Busson, A. Cappati, C. Charlot, F. Damas, O. Davignon, B. Diab, G. Falmagne, B. A. Fontana Santos Alves, S. Ghosh, R. Granier de Cassagnac, A. Hakimi, B. Harikrishnan, G. Liu, J. Motta, M. Nguyen, C. Ochando, L. Portales, R. Salerno, U. Sarkar, J. B. Sauvan, Y. Sirois, A. Tarabini, E. Vernazza, A. Zabi, A. Zghiche, J.-L. Agram, J. Andrea, D. Apparu, D. Bloch, G. Bourgatte, J.-M. Brom, E. C. Chabert, C. Collard, D. Darej, U. Goerlach, C. Grimault, A.-C. Le Bihan, P. Van Hove, S. Beauceron, B. Blancon, G. Boudoul, A. Carle, N. Chanon, J. Choi, D. Contardo, P. Depasse, C. Dozen, H. El Mamouni, J. Fay, S. Gascon, M. Gouzevitch, G. Grenier, B. Ille, I. B. Laktineh, M. Lethuillier, L. Mirabito, S. Perries, L. Torterotot, M. Vander Donckt, P. Verdier, S. Viret, D. Lomidze, I. Lomidze, Z. Tsamalaidze, V. Botta, L. Feld, K. Klein, M. Lipinski, D. Meuser, A. Pauls, N. Röwert, M. Teroerde, S. Diekmann, A. Dodonova, N. Eich, D. Eliseev, M. Erdmann, P. Fackeldey, D. Fasanella, B. Fischer, T. Hebbeker, K. Hoepfner, F. Ivone, M. Y. Lee, L. Mastrolorenzo, M. Merschmeyer, A. Meyer, S. Mondal, S. Mukherjee, D. Noll, A. Novak, F. Nowotny, A. Pozdnyakov, Y. Rath, W. Redjeb, F. Rehm, H. Reithler, A. Schmidt, S. C. Schuler, A. Sharma, A. Stein, F. Torres Da Silva De Araujo, L. Vigilante, S. Wiedenbeck, S. Zaleski, C. Dziwok, G. Flügge, W. Haj Ahmad, O. Hlushchenko, T. Kress, A. Nowack, O. Pooth, A. Stahl, T. Ziemons, A. Zotz, H. Aarup Petersen, M. Aldaya Martin, J. Alimena, P. Asmuss, S. Baxter, M. Bayatmakou, H. Becerril Gonzalez, O. Behnke, S. Bhattacharya, F. Blekman, K. Borras, D. Brunner, A. Campbell, A. Cardini, C. Cheng, F. Colombina, S. Consuegra Rodríguez, G. Correia Silva, M. De Silva, G. Eckerlin, D. Eckstein, L. I. Estevez Banos, O. Filatov, E. Gallo, A. Geiser, A. Giraldi, G. Greau, A. Grohsjean, V. Guglielmi, M. Guthoff, A. Jafari, N. Z. Jomhari, B. Kaech, M. Kasemann, H. Kaveh, C. Kleinwort, R. Kogler, M. Komm, D. Krücker, W. Lange, D. Leyva Pernia, K. Lipka, W. Lohmann, R. Mankel, I.-A. Melzer-Pellmann, M. Mendizabal Morentin, J. Metwally, A. B. Meyer, G. Milella, M. Mormile, A. Mussgiller, A. Nürnberg, Y. Otarid, D. Pérez Adán, E. Ranken, A. Raspereza, B. Ribeiro Lopes, J. Rübenach, A. Saggio, M. Savitskyi, M. Scham, V. Scheurer, S. Schnake, P. Schütze, C. Schwanenberger, M. Shchedrolosiev, R. E. Sosa Ricardo, D. Stafford, N. Tonon, M. Van De Klundert, F. Vazzoler, A. Ventura Barroso, R. Walsh, D. Walter, Q. Wang, Y. Wen, K. Wichmann, L. Wiens, C. Wissing, S. Wuchterl, Y. Yang, A. Zimermmane Castro Santos, A. Albrecht, S. Albrecht, M. Antonello, S. Bein, L. Benato, M. Bonanomi, P. Connor, K. De Leo, M. Eich, K. El Morabit, F. Feindt, A. Fröhlich, C. Garbers, E. Garutti, M. Hajheidari, J. Haller, A. Hinzmann, H. R. Jabusch, G. Kasieczka, P. Keicher, R. Klanner, W. Korcari, T. Kramer, V. Kutzner, F. Labe, J. Lange, A. Lobanov, C. Matthies, A. Mehta, L. Moureaux, M. Mrowietz, A. Nigamova, Y. Nissan, A. Paasch, K. J. Pena Rodriguez, T. Quadfasel, M. Rieger, D. Savoiu, J. Schindler, P. Schleper, M. Schröder, J. Schwandt, M. Sommerhalder, H. Stadie, G. Steinbrück, A. Tews, M. Wolf, S. Brommer, M. Burkart, E. Butz, T. Chwalek, A. Dierlamm, A. Droll, N. Faltermann, M. Giffels, J. O. Gosewisch, A. Gottmann, F. Hartmann, M. Horzela, U. Husemann, M. Klute, R. Koppenhöfer, M. Link, A. Lintuluoto, S. Maier, S. Mitra, Th. Müller, M. Neukum, M. Oh, G. Quast, K. Rabbertz, I. Shvetsov, H.J. Simonis, N. Trevisani, R. Ulrich, J. van der Linden, R. F. Von Cube, M. Wassmer, S. Wieland, R. Wolf, S. Wozniewski, S. Wunsch, X. Zuo, G. Anagnostou, P. Assiouras, G. Daskalakis, A. Kyriakis, D. Loukas, A. Stakia, M. Diamantopoulou, D. Karasavvas, P. Kontaxakis, A. Manousakis-Katsikakis, A. Panagiotou, I. Papavergou, N. Saoulidou, K. Theofilatos, E. Tziaferi, K. Vellidis, I. Zisopoulos, G. Bakas, T. Chatzistavrou, G. Karapostoli, K. Kousouris, I. Papakrivopoulos, G. Tsipolitis, A. Zacharopoulou, K. Adamidis, I. Bestintzanos, I. Evangelou, C. Foudas, P. Gianneios, C. Kamtsikis, P. Katsoulis, P. Kokkas, P. G. Kosmoglou Kioseoglou, N. Manthos, I. Papadopoulos, J. Strologas, M. Csanád, K. Farkas, M. M. A. Gadallah, P. Major, K. Mandal, G. Pásztor, A. J. Rádl, O. Surányi, G. I. Veres, M. Bartók, G. Bencze, C. Hajdu, D. Horvath, F. Sikler, V. Veszpremi, N. Beni, S. Czellar, J. Karancsi, J. Molnar, Z. Szillasi, D. Teyssier, P. Raics, B. Ujvari, G. Zilizi, T. Csorgo, F. Nemes, T. Novak, J. Babbar, S. Bansal, S. B. Beri, V. Bhatnagar, G. Chaudhary, S. Chauhan, N. Dhingra, R. Gupta, A. Kaur, A. Kaur, H. Kaur, M. Kaur, S. Kumar, P. Kumari, M. Meena, K. Sandeep, T. Sheokand, J. B. Singh, A. Singla, A. Ahmed, A. Bhardwaj, A. Chhetri, B. C. Choudhary, A. Kumar, M. Naimuddin, K. Ranjan, S. Saumya, S. Baradia, S. Barman, S. Bhattacharya, D. Bhowmik, S. Dutta, S. Dutta, B. Gomber, M. Maity, P. Palit, G. Saha, B. Sahu, S. Sarkar, P. K. Behera, S. C. Behera, S. Chatterjee, P. Kalbhor, J. R. Komaragiri, D. Kumar, A. Muhammad, L. Panwar, R. Pradhan, P. R. Pujahari, N. R. Saha, A. Sharma, A. K. Sikdar, S. Verma, K. Naskar, T. Aziz, I. Das, S. Dugad, M. Kumar, G. B. Mohanty, P. Suryadevara, S. Banerjee, M. Guchait, S. Karmakar, S. Kumar, G. Majumder, K. Mazumdar, S. Mukherjee, A. Thachayath, S. Bahinipati, A. K. Das, C. Kar, P. Mal, T. Mishra, V. K. Muraleedharan Nair Bindhu, A. Nayak, P. Saha, S. K. Swain, D. Vats, A. Alpana, S. Dube, B. Kansal, A. Laha, S. Pandey, A. Rastogi, S. Sharma, H. Bakhshiansohi, E. Khazaie, M. Zeinali, S. Chenarani, S. M. Etesami, M. Khakzad, M. Mohammadi Najafabadi, M. Grunewald, M. Abbrescia, R. Aly, C. Aruta, A. Colaleo, D. Creanza, L. Cristella, N. De Filippis, M. De Palma, A. Di Florio, W. Elmetenawee, F. Errico, L. Fiore, G. Iaselli, G. Maggi, M. Maggi, I. Margjeka, V. Mastrapasqua, S. My, S. Nuzzo, A. Pellecchia, A. Pompili, G. Pugliese, R. Radogna, D. Ramos, A. Ranieri, G. Selvaggi, L. Silvestris, F. M. Simone, Ü. Sözbilir, A. Stamerra, R. Venditti, P. Verwilligen, G. Abbiendi, C. Battilana, D. Bonacorsi, L. Borgonovi, L. Brigliadori, R. Campanini, P. Capiluppi, A. Castro, F. R. Cavallo, M. Cuffiani, G. M. Dallavalle, T. Diotalevi, F. Fabbri, A. Fanfani, P. Giacomelli, L. Giommi, C. Grandi, L. Guiducci, S. Lo Meo, L. Lunerti, S. Marcellini, G. Masetti, F. L. Navarria, A. Perrotta, F. Primavera, A. M. Rossi, T. Rovelli, G. P. Siroli, S. Costa, A. Di Mattia, R. Potenza, A. Tricomi, C. Tuve, G. Barbagli, G. Bardelli, B. Camaiani, A. Cassese, R. Ceccarelli, V. Ciulli, C. Civinini, R. D’Alessandro, E. Focardi, G. Latino, P. Lenzi, M. Lizzo, M. Meschini, S. Paoletti, G. Sguazzoni, L. Viliani, L. Benussi, S. Bianco, S. Meola, D. Piccolo, M. Bozzo, P. Chatagnon, F. Ferro, E. Robutti, S. Tosi, A. Benaglia, G. Boldrini, F. Brivio, F. Cetorelli, F. De Guio, M. E. Dinardo, P. Dini, S. Gennai, A. Ghezzi, P. Govoni, L. Guzzi, M. T. Lucchini, M. Malberti, S. Malvezzi, A. Massironi, D. Menasce, L. Moroni, M. Paganoni, D. Pedrini, B. S. Pinolini, S. Ragazzi, N. Redaelli, T. Tabarelli de Fatis, D. Zuolo, S. Buontempo, F. Carnevali, N. Cavallo, A. De Iorio, F. Fabozzi, A. O. M. Iorio, L. Lista, P. Paolucci, B. Rossi, C. Sciacca, P. Azzi, N. Bacchetta, P. Bortignon, A. Bragagnolo, R. Carlin, P. Checchia, T. Dorigo, F. Gasparini, U. Gasparini, G. Grosso, L. Layer, E. Lusiani, M. Margoni, G. Maron, A. T. Meneguzzo, J. Pazzini, P. Ronchese, R. Rossin, F. Simonetto, G. Strong, M. Tosi, H. Yarar, M. Zanetti, P. Zotto, A. Zucchetta, G. Zumerle, S. Abu Zeid, C. Aimè, A. Braghieri, S. Calzaferri, D. Fiorina, P. Montagna, V. Re, C. Riccardi, P. Salvini, I. Vai, P. Vitulo, P. Asenov, G. M. Bilei, D. Ciangottini, L. Fanò, M. Magherini, G. Mantovani, V. Mariani, M. Menichelli, F. Moscatelli, A. Piccinelli, M. Presilla, A. Rossi, A. Santocchia, D. Spiga, T. Tedeschi, P. Azzurri, G. Bagliesi, V. Bertacchi, R. Bhattacharya, L. Bianchini, T. Boccali, E. Bossini, D. Bruschini, R. Castaldi, M. A. Ciocci, V. D’Amante, R. Dell’Orso, S. Donato, A. Giassi, F. Ligabue, D. Matos Figueiredo, A. Messineo, M. Musich, F. Palla, S. Parolia, G. Ramirez-Sanchez, A. Rizzi, G. Rolandi, S. Roy Chowdhury, T. Sarkar, A. Scribano, P. Spagnolo, R. Tenchini, G. Tonelli, N. Turini, A. Venturi, P. G. Verdini, P. Barria, M. Campana, F. Cavallari, D. Del Re, E. Di Marco, M. Diemoz, E. Longo, P. Meridiani, G. Organtini, F. Pandolfi, R. Paramatti, C. Quaranta, S. Rahatlou, C. Rovelli, F. Santanastasio, L. Soffi, R. Tramontano, N. Amapane, R. Arcidiacono, S. Argiro, M. Arneodo, N. Bartosik, R. Bellan, A. Bellora, C. Biino, N. Cartiglia, M. Costa, R. Covarelli, N. Demaria, M. Grippo, B. Kiani, F. Legger, F. Luongo, C. Mariotti, S. Maselli, A. Mecca, E. Migliore, M. Monteno, R. Mulargia, M. M. Obertino, G. Ortona, L. Pacher, N. Pastrone, M. Pelliccioni, M. Ruspa, K. Shchelina, F. Siviero, V. Sola, A. Solano, D. Soldi, A. Staiano, C. Tarricone, M. Tornago, D. Trocino, G. Umoret, A. Vagnerini, E. Vlasov, S. Belforte, V. Candelise, M. Casarsa, F. Cossutti, G. Della Ricca, G. Sorrentino, S. Dogra, C. Huh, B. Kim, D. H. Kim, G. N. Kim, J. Kim, J. Lee, S. W. Lee, C. S. Moon, Y. D. Oh, S. I. Pak, M. S. Ryu, S. Sekmen, Y. C. Yang, H. Kim, D. H. Moon, E. Asilar, T. J. Kim, J. Park, S. Choi, S. Han, B. Hong, K. Lee, K. S. Lee, J. Lim, J. Park, S. K. Park, J. Yoo, J. Goh, H. S. Kim, Y. Kim, S. Lee, J. Almond, J. H. Bhyun, J. Choi, S. Jeon, J. Kim, J. S. Kim, S. Ko, H. Kwon, H. Lee, S. Lee, B. H. Oh, S. B. Oh, H. Seo, U. K. Yang, I. Yoon, W. Jang, D. Y. Kang, Y. Kang, D. Kim, S. Kim, B. Ko, J. S. H. Lee, Y. Lee, J. A. Merlin, I. C. Park, Y. Roh, D. Song, I. J. Watson, S. Yang, S. Ha, H. D. Yoo, M. Choi, M. R. Kim, H. Lee, Y. Lee, I. Yu, T. Beyrouthy, Y. Maghrbi, K. Dreimanis, G. Pikurs, A. Potrebko, M. Seidel, V. Veckalns, M. Ambrozas, A. Carvalho Antunes De Oliveira, A. Juodagalvis, A. Rinkevicius, G. Tamulaitis, N. Bin Norjoharuddeen, S. Y. Hoh, I. Yusuff, Z. Zolkapli, J. F. Benitez, A. Castaneda Hernandez, H. A. Encinas Acosta, L. G. Gallegos Maríñez, M. León Coello, J. A. Murillo Quijada, A. Sehrawat, L. Valencia Palomo, G. Ayala, H. Castilla-Valdez, I. Heredia-De La Cruz, R. Lopez-Fernandez, C. A. Mondragon Herrera, D. A. Perez Navarro, A. Sánchez Hernández, C. Oropeza Barrera, F. Vazquez Valencia, I. Pedraza, H. A. Salazar Ibarguen, C. Uribe Estrada, I. Bubanja, J. Mijuskovic, N. Raicevic, A. Ahmad, M. I. Asghar, A. Awais, M. I. M. Awan, M. Gul, H. R. Hoorani, W. A. Khan, V. Avati, L. Grzanka, M. Malawski, H. Bialkowska, M. Bluj, B. Boimska, M. Górski, M. Kazana, M. Szleper, P. Zalewski, K. Bunkowski, K. Doroba, A. Kalinowski, M. Konecki, J. Krolikowski, M. Araujo, P. Bargassa, D. Bastos, A. Boletti, P. Faccioli, M. Gallinaro, J. Hollar, N. Leonardo, T. Niknejad, M. Pisano, J. Seixas, J. Varela, P. Adzic, M. Dordevic, P. Milenovic, J. Milosevic, M. Aguilar-Benitez, J. Alcaraz Maestre, M. Barrio Luna, Cristina F. Bedoya, M. Cepeda, M. Cerrada, N. Colino, B. De La Cruz, A. Delgado Peris, D. Fernández Del Val, J. P. Fernández Ramos, J. Flix, M. C. Fouz, O. Gonzalez Lopez, S. Goy Lopez, J. M. Hernandez, M. I. Josa, J. León Holgado, D. Moran, C. Perez Dengra, A. Pérez-Calero Yzquierdo, J. Puerta Pelayo, I. Redondo, D. D. Redondo Ferrero, L. Romero, S. Sánchez Navas, J. Sastre, L. Urda Gómez, J. Vazquez Escobar, C. Willmott, J. F. de Trocóniz, B. Alvarez Gonzalez, J. Cuevas, J. Fernandez Menendez, S. Folgueras, I. Gonzalez Caballero, J. R. González Fernández, E. Palencia Cortezon, C. Ramón Álvarez, V. Rodríguez Bouza, A. Soto Rodríguez, A. Trapote, C. Vico Villalba, J. A. Brochero Cifuentes, I. J. Cabrillo, A. Calderon, J. Duarte Campderros, M. Fernandez, C. Fernandez Madrazo, A. García Alonso, G. Gomez, C. Lasaosa García, C. Martinez Rivero, P. Martinez Ruiz del Arbol, F. Matorras, P. Matorras Cuevas, J. Piedra Gomez, C. Prieels, L. Scodellaro, I. Vila, J. M. Vizan Garcia, M. K. Jayananda, B. Kailasapathy, D. U. J. Sonnadara, D. D. C. Wickramarathna, W. G. D. Dharmaratna, K. Liyanage, N. Perera, N. Wickramage, D. Abbaneo, E. Auffray, G. Auzinger, J. Baechler, P. Baillon, D. Barney, J. Bendavid, A. Bermúdez Martínez, M. Bianco, B. Bilin, A. A. Bin Anuar, A. Bocci, E. Brondolin, C. Caillol, T. Camporesi, G. Cerminara, N. Chernyavskaya, S. S. Chhibra, S. Choudhury, M. Cipriani, D. d’Enterria, A. Dabrowski, A. David, A. De Roeck, M. M. Defranchis, M. Deile, M. Dobson, M. Dünser, N. Dupont, F. Fallavollita, A. Florent, L. Forthomme, G. Franzoni, W. Funk, S. Ghosh, S. Giani, D. Gigi, K. Gill, F. Glege, L. Gouskos, E. Govorkova, M. Haranko, J. Hegeman, V. Innocente, T. James, P. Janot, J. Kaspar, J. Kieseler, N. Kratochwil, S. Laurila, P. Lecoq, E. Leutgeb, C. Lourenço, B. Maier, L. Malgeri, M. Mannelli, A. C. Marini, F. Meijers, S. Mersi, E. Meschi, F. Moortgat, M. Mulders, S. Orfanelli, L. Orsini, F. Pantaleo, E. Perez, M. Peruzzi, A. Petrilli, G. Petrucciani, A. Pfeiffer, M. Pierini, D. Piparo, M. Pitt, H. Qu, T. Quast, D. Rabady, A. Racz, G. Reales Gutiérrez, M. Rovere, H. Sakulin, J. Salfeld-Nebgen, S. Scarfi, M. Selvaggi, A. Sharma, P. Silva, P. Sphicas, A. G. Stahl Leiton, S. Summers, K. Tatar, D. Treille, P. Tropea, A. Tsirou, J. Wanczyk, K. A. Wozniak, W. D. Zeuner, L. Caminada, A. Ebrahimi, W. Erdmann, R. Horisberger, Q. Ingram, H. C. Kaestli, D. Kotlinski, C. Lange, M. Missiroli, L. Noehte, T. Rohe, T. K. Aarrestad, K. Androsov, M. Backhaus, A. Calandri, K. Datta, A. De Cosa, G. Dissertori, M. Dittmar, M. Donegà, F. Eble, M. Galli, K. Gedia, F. Glessgen, T. A. Gómez Espinosa, C. Grab, D. Hits, W. Lustermann, A.-M. Lyon, R. A. Manzoni, L. Marchese, C. Martin Perez, A. Mascellani, F. Nessi-Tedaldi, J. Niedziela, F. Pauss, V. Perovic, S. Pigazzini, M. G. Ratti, M. Reichmann, C. Reissel, T. Reitenspiess, B. Ristic, F. Riti, D. Ruini, D. A. Sanz Becerra, R. Seidita, J. Steggemann, D. Valsecchi, R. Wallny, C. Amsler, P. Bärtschi, C. Botta, D. Brzhechko, M. F. Canelli, K. Cormier, A. De Wit, R. Del Burgo, J. K. Heikkilä, M. Huwiler, W. Jin, A. Jofrehei, B. Kilminster, S. Leontsinis, S. P. Liechti, A. Macchiolo, P. Meiring, V. M. Mikuni, U. Molinatti, I. Neutelings, A. Reimers, P. Robmann, S. Sanchez Cruz, K. Schweiger, M. Senger, Y. Takahashi, C. Adloff, C. M. Kuo, W. Lin, P. K. Rout, P. C. Tiwari, S. S. Yu, L. Ceard, Y. Chao, K. F. Chen, P. s. Chen, H. Cheng, W.-S. Hou, R. Khurana, G. Kole, Y. y. Li, R.-S. Lu, E. Paganis, A. Psallidas, A. Steen, H. Y. Wu, E. Yazgan, C. Asawatangtrakuldee, N. Srimanobhas, V. Wachirapusitanand, D. Agyel, F. Boran, Z. S. Demiroglu, F. Dolek, I. Dumanoglu, E. Eskut, Y. Guler, E. Gurpinar Guler, C. Isik, O. Kara, A. Kayis Topaksu, U. Kiminsu, G. Onengut, K. Ozdemir, A. Polatoz, A. E. Simsek, B. Tali, U. G. Tok, S. Turkcapar, E. Uslan, I. S. Zorbakir, G. Karapinar, K. Ocalan, M. Yalvac, B. Akgun, I. O. Atakisi, E. Gülmez, M. Kaya, O. Kaya, S. Tekten, A. Cakir, K. Cankocak, Y. Komurcu, S. Sen, O. Aydilek, S. Cerci, B. Hacisahinoglu, I. Hos, B. Isildak, B. Kaynak, S. Ozkorucuklu, C. Simsek, D. Sunar Cerci, B. Grynyov, L. Levchuk, D. Anthony, J. J. Brooke, A. Bundock, E. Clement, D. Cussans, H. Flacher, M. Glowacki, J. Goldstein, H. F. Heath, L. Kreczko, B. Krikler, S. Paramesvaran, S. Seif El Nasr-Storey, V. J. Smith, N. Stylianou, K. Walkingshaw Pass, R. White, A. H. Ball, K. W. Bell, A. Belyaev, C. Brew, R. M. Brown, D. J. A. Cockerill, C. Cooke, K. V. Ellis, K. Harder, S. Harper, M.-L. Holmberg, Sh. Jain, J. Linacre, K. Manolopoulos, D. M. Newbold, E. Olaiya, D. Petyt, T. Reis, G. Salvi, T. Schuh, C. H. Shepherd-Themistocleous, I. R. Tomalin, T. Williams, R. Bainbridge, P. Bloch, S. Bonomally, J. Borg, C. E. Brown, O. Buchmuller, V. Cacchio, C. A. Carrillo Montoya, V. Cepaitis, G. S. Chahal, D. Colling, J. S. Dancu, P. Dauncey, G. Davies, J. Davies, M. Della Negra, S. Fayer, G. Fedi, G. Hall, M. H. Hassanshahi, A. Howard, G. Iles, J. Langford, L. Lyons, A.-M. Magnan, S. Malik, A. Martelli, M. Mieskolainen, D. G. Monk, J. Nash, M. Pesaresi, B. C. Radburn-Smith, D. M. Raymond, A. Richards, A. Rose, E. Scott, C. Seez, R. Shukla, A. Tapper, K. Uchida, G. P. Uttley, L. H. Vage, T. Virdee, M. Vojinovic, N. Wardle, S. N. Webb, D. Winterbottom, K. Coldham, J. E. Cole, A. Khan, P. Kyberd, I. D. Reid, S. Abdullin, A. Brinkerhoff, B. Caraway, J. Dittmann, K. Hatakeyama, A. R. Kanuganti, B. McMaster, M. Saunders, S. Sawant, C. Sutantawibul, M. Toms, J. Wilson, R. Bartek, A. Dominguez, C. Huerta Escamilla, R. Uniyal, A. M. Vargas Hernandez, R. Chudasama, S. I. Cooper, D. Di Croce, S. V. Gleyzer, C. U. Perez, P. Rumerio, E. Usai, C. West, A. Akpinar, A. Albert, D. Arcaro, C. Cosby, Z. Demiragli, C. Erice, E. Fontanesi, D. Gastler, S. May, J. Rohlf, K. Salyer, D. Sperka, D. Spitzbart, I. Suarez, A. Tsatsos, S. Yuan, G. Benelli, X. Coubez, D. Cutts, M. Hadley, U. Heintz, J. M. Hogan, T. Kwon, G. Landsberg, K. T. Lau, D. Li, J. Luo, M. Narain, N. Pervan, S. Sagir, F. Simpson, W. Y. Wong, X. Yan, D. Yu, W. Zhang, S. Abbott, J. Bonilla, C. Brainerd, R. Breedon, M. Calderon De La Barca Sanchez, M. Chertok, J. Conway, P. T. Cox, R. Erbacher, G. Haza, F. Jensen, O. Kukral, G. Mocellin, M. Mulhearn, D. Pellett, B. Regnery, Y. Yao, F. Zhang, M. Bachtis, R. Cousins, A. Datta, J. Hauser, M. Ignatenko, M.A. Iqbal, T. Lam, E. Manca, W. A. Nash, D. Saltzberg, B. Stone, V. Valuev, R. Clare, J. W. Gary, M. Gordon, G. Hanson, O. R. Long, N. Manganelli, W. Si, S. Wimpenny, J. G. Branson, S. Cittolin, S. Cooperstein, D. Diaz, J. Duarte, R. Gerosa, L. Giannini, J. Guiang, R. Kansal, V. Krutelyov, R. Lee, J. Letts, M. Masciovecchio, F. Mokhtar, M. Pieri, M. Quinnan, B. V. Sathia Narayanan, V. Sharma, M. Tadel, E. Vourliotis, F. Würthwein, Y. Xiang, A. Yagil, C. Campagnari, M. Citron, G. Collura, A. Dorsett, J. Incandela, M. Kilpatrick, J. Kim, A. J. Li, P. Masterson, H. Mei, M. Oshiro, J. Richman, U. Sarica, R. Schmitz, F. Setti, J. Sheplock, P. Siddireddy, D. Stuart, S. Wang, A. Bornheim, O. Cerri, I. Dutta, A. Latorre, J. M. Lawhorn, J. Mao, H. B. Newman, T. Q. Nguyen, M. Spiropulu, J. R. Vlimant, C. Wang, S. Xie, R. Y. Zhu, J. Alison, S. An, M. B. Andrews, P. Bryant, V. Dutta, T. Ferguson, A. Harilal, C. Liu, T. Mudholkar, S. Murthy, M. Paulini, A. Roberts, A. Sanchez, W. Terrill, J. P. Cumalat, W. T. Ford, A. Hassani, G. Karathanasis, E. MacDonald, F. Marini, A. Perloff, C. Savard, N. Schonbeck, K. Stenson, K. A. Ulmer, S. R. Wagner, N. Zipper, J. Alexander, S. Bright-Thonney, X. Chen, D. J. Cranshaw, J. Fan, X. Fan, D. Gadkari, S. Hogan, J. Monroy, J. R. Patterson, J. Reichert, M. Reid, A. Ryd, J. Thom, P. Wittich, R. Zou, M. Albrow, M. Alyari, G. Apollinari, A. Apresyan, L. A. T. Bauerdick, D. Berry, J. Berryhill, P. C. Bhat, K. Burkett, J. N. Butler, A. Canepa, G. B. Cerati, H. W. K. Cheung, F. Chlebana, K. F. Di Petrillo, J. Dickinson, V. D. Elvira, Y. Feng, J. Freeman, A. Gandrakota, Z. Gecse, L. Gray, D. Green, S. Grünendahl, D. Guerrero, O. Gutsche, R. M. Harris, R. Heller, T. C. Herwig, J. Hirschauer, L. Horyn, B. Jayatilaka, S. Jindariani, M. Johnson, U. Joshi, T. Klijnsma, B. Klima, K. H. M. Kwok, S. Lammel, D. Lincoln, R. Lipton, T. Liu, C. Madrid, K. Maeshima, C. Mantilla, D. Mason, P. McBride, P. Merkel, S. Mrenna, S. Nahn, J. Ngadiuba, D. Noonan, S. Norberg, V. Papadimitriou, N. Pastika, K. Pedro, C. Pena, F. Ravera, A. Reinsvold Hall, L. Ristori, E. Sexton-Kennedy, N. Smith, A. Soha, L. Spiegel, J. Strait, L. Taylor, S. Tkaczyk, N. V. Tran, L. Uplegger, E. W. Vaandering, I. Zoi, P. Avery, D. Bourilkov, L. Cadamuro, P. Chang, V. Cherepanov, R. D. Field, E. Koenig, M. Kolosova, J. Konigsberg, A. Korytov, E. Kuznetsova, K. H. Lo, K. Matchev, N. Menendez, G. Mitselmakher, A. Muthirakalayil Madhu, N. Rawal, D. Rosenzweig, S. Rosenzweig, K. Shi, J. Wang, Z. Wu, T. Adams, A. Askew, N. Bower, R. Habibullah, V. Hagopian, T. Kolberg, G. Martinez, H. Prosper, O. Viazlo, M. Wulansatiti, R. Yohay, J. Zhang, M. M. Baarmand, S. Butalla, T. Elkafrawy, M. Hohlmann, R. Kumar Verma, M. Rahmani, F. Yumiceva, M. R. Adams, R. Cavanaugh, S. Dittmer, O. Evdokimov, C. E. Gerber, D. J. Hofman, D. S. Lemos, A. H. Merrit, C. Mills, G. Oh, T. Roy, S. Rudrabhatla, M. B. Tonjes, N. Varelas, X. Wang, Z. Ye, J. Yoo, M. Alhusseini, K. Dilsiz, L. Emediato, G. Karaman, O. K. Köseyan, J.-P. Merlo, A. Mestvirishvili, J. Nachtman, O. Neogi, H. Ogul, Y. Onel, A. Penzo, C. Snyder, E. Tiras, O. Amram, B. Blumenfeld, L. Corcodilos, J. Davis, A. V. Gritsan, S. Kyriacou, P. Maksimovic, J. Roskes, S. Sekhar, M. Swartz, T.Á. Vámi, A. Abreu, L. F. Alcerro Alcerro, J. Anguiano, P. Baringer, A. Bean, Z. Flowers, J. King, G. Krintiras, M. Lazarovits, C. Le Mahieu, C. Lindsey, J. Marquez, N. Minafra, M. Murray, M. Nickel, C. Rogan, C. Royon, R. Salvatico, S. Sanders, C. Smith, Q. Wang, G. Wilson, B. Allmond, S. Duric, A. Ivanov, K. Kaadze, A. Kalogeropoulos, D. Kim, Y. Maravin, T. Mitchell, A. Modak, K. Nam, D. Roy, F. Rebassoo, D. Wright, E. Adams, A. Baden, O. Baron, A. Belloni, A. Bethani, S. C. Eno, N. J. Hadley, S. Jabeen, R. G. Kellogg, T. Koeth, Y. Lai, S. Lascio, A. C. Mignerey, S. Nabili, C. Palmer, C. Papageorgakis, L. Wang, K. Wong, W. Busza, I. A. Cali, Y. Chen, M. D’Alfonso, J. Eysermans, C. Freer, G. Gomez-Ceballos, M. Goncharov, P. Harris, D. Kovalskyi, J. Krupa, Y.-J. Lee, K. Long, C. Mironov, C. Paus, D. Rankin, C. Roland, G. Roland, Z. Shi, G. S. F. Stephans, J. Wang, Z. Wang, B. Wyslouch, T. J. Yang, R. M. Chatterjee, B. Crossman, J. Hiltbrand, B. M. Joshi, C. Kapsiak, M. Krohn, Y. Kubota, D. Mahon, J. Mans, M. Revering, R. Rusack, R. Saradhy, N. Schroeder, N. Strobbe, M. A. Wadud, L. M. Cremaldi, K. Bloom, M. Bryson, D. R. Claes, C. Fangmeier, L. Finco, F. Golf, C. Joo, R. Kamalieddin, I. Kravchenko, I. Reed, J. E. Siado, G. R. Snow, W. Tabb, A. Wightman, F. Yan, A. G. Zecchinelli, G. Agarwal, H. Bandyopadhyay, L. Hay, I. Iashvili, A. Kharchilava, C. McLean, M. Morris, D. Nguyen, J. Pekkanen, S. Rappoccio, A. Williams, G. Alverson, E. Barberis, Y. Haddad, Y. Han, A. Krishna, J. Li, J. Lidrych, G. Madigan, B. Marzocchi, D. M. Morse, V. Nguyen, T. Orimoto, A. Parker, L. Skinnari, A. Tishelman-Charny, T. Wamorkar, B. Wang, A. Wisecarver, D. Wood, S. Bhattacharya, J. Bueghly, Z. Chen, A. Gilbert, K. A. Hahn, Y. Liu, N. Odell, M. H. Schmitt, M. Velasco, R. Band, R. Bucci, M. Cremonesi, A. Das, R. Goldouzian, M. Hildreth, K. Hurtado Anampa, C. Jessop, K. Lannon, J. Lawrence, N. Loukas, L. Lutton, J. Mariano, N. Marinelli, I. Mcalister, T. McCauley, C. Mcgrady, K. Mohrman, C. Moore, Y. Musienko, R. Ruchti, A. Townsend, M. Wayne, H. Yockey, M. Zarucki, L. Zygala, B. Bylsma, M. Carrigan, L. S. Durkin, C. Hill, M. Joyce, A. Lesauvage, M. Nunez Ornelas, K. Wei, B. L. Winer, B. R. Yates, F. M. Addesa, P. Das, G. Dezoort, P. Elmer, A. Frankenthal, B. Greenberg, N. Haubrich, S. Higginbotham, G. Kopp, S. Kwan, D. Lange, A. Loeliger, D. Marlow, I. Ojalvo, J. Olsen, D. Stickland, C. Tully, S. Malik, A. S. Bakshi, V. E. Barnes, R. Chawla, S. Das, L. Gutay, M. Jones, A. W. Jung, D. Kondratyev, A. M. Koshy, M. Liu, G. Negro, N. Neumeister, G. Paspalaki, S. Piperov, A. Purohit, J. F. Schulte, M. Stojanovic, J. Thieman, A. K. Virdi, F. Wang, R. Xiao, W. Xie, J. Dolen, N. Parashar, D. Acosta, A. Baty, T. Carnahan, S. Dildick, K. M. Ecklund, P. J. Fernández Manteca, S. Freed, P. Gardner, F. J. M. Geurts, A. Kumar, W. Li, B. P. Padley, R. Redjimi, J. Rotter, S. Yang, E. Yigitbasi, Y. Zhang, A. Bodek, P. de Barbaro, R. Demina, J. L. Dulemba, C. Fallon, A. Garcia-Bellido, O. Hindrichs, A. Khukhunaishvili, P. Parygin, E. Popova, R. Taus, G. P. Van Onsem, K. Goulianos, B. Chiarito, J. P. Chou, Y. Gershtein, E. Halkiadakis, A. Hart, M. Heindl, D. Jaroslawski, O. Karacheban, I. Laflotte, A. Lath, R. Montalvo, K. Nash, M. Osherson, H. Routray, S. Salur, S. Schnetzer, S. Somalwar, R. Stone, S. A. Thayil, S. Thomas, H. Wang, H. Acharya, A. G. Delannoy, S. Fiorendi, T. Holmes, E. Nibigira, S. Spanier, O. Bouhali, M. Dalchenko, A. Delgado, R. Eusebi, J. Gilmore, T. Huang, T. Kamon, H. Kim, S. Luo, S. Malhotra, R. Mueller, D. Overton, D. Rathjens, A. Safonov, N. Akchurin, J. Damgov, V. Hegde, K. Lamichhane, S. W. Lee, T. Mengke, S. Muthumuni, T. Peltola, I. Volobouev, A. Whitbeck, E. Appelt, S. Greene, A. Gurrola, W. Johns, A. Melo, F. Romeo, P. Sheldon, S. Tuo, J. Velkovska, J. Viinikainen, B. Cardwell, B. Cox, G. Cummings, J. Hakala, R. Hirosky, A. Ledovskoy, A. Li, C. Neu, C. E. Perez Lara, P. E. Karchin, A. Aravind, S. Banerjee, K. Black, T. Bose, S. Dasu, I. De Bruyn, P. Everaerts, C. Galloni, H. He, M. Herndon, A. Herve, C. K. Koraka, A. Lanaro, R. Loveless, J. Madhusudanan Sreekala, A. Mallampalli, A. Mohammadi, S. Mondal, G. Parida, D. Pinna, A. Savin, V. Shang, V. Sharma, W. H. Smith, D. Teague, H. F. Tsoi, W. Vetens, A. Warden, S. Afanasiev, V. Andreev, Yu. Andreev, T. Aushev, M. Azarkin, A. Babaev, A. Belyaev, V. Blinov, E. Boos, V. Borshch, D. Budkouski, V. Bunichev, M. Chadeeva, V. Chekhovsky, M. Danilov, A. Dermenev, T. Dimova, I. Dremin, M. Dubinin, L. Dudko, V. Epshteyn, A. Ershov, G. Gavrilov, V. Gavrilov, S. Gninenko, V. Golovtcov, N. Golubev, I. Golutvin, I. Gorbunov, Y. Ivanov, V. Kachanov, L. Kardapoltsev, V. Karjavine, A. Karneyeu, V. Kim, M. Kirakosyan, D. Kirpichnikov, M. Kirsanov, V. Klyukhin, O. Kodolova, D. Konstantinov, V. Korenkov, A. Kozyrev, N. Krasnikov, A. Lanev, P. Levchenko, A. Litomin, N. Lychkovskaya, V. Makarenko, A. Malakhov, V. Matveev, V. Murzin, A. Nikitenko, S. Obraztsov, A. Oskin, I. Ovtin, V. Palichik, V. Perelygin, M. Perfilov, S. Petrushanko, V. Popov, O. Radchenko, V. Rusinov, M. Savina, V. Savrin, V. Shalaev, S. Shmatov, S. Shulha, Y. Skovpen, S. Slabospitskii, V. Smirnov, D. Sosnov, V. Sulimov, E. Tcherniaev, A. Terkulov, O. Teryaev, I. Tlisova, A. Toropin, L. Uvarov, A. Uzunian, A. Vorobyev, N. Voytishin, B. S. Yuldashev, A. Zarubin, I. Zhizhin, A. Zhokin, CMS Collaboration

**Affiliations:** 1 Yerevan Physics Institute, Yerevan, Armenia; 2 Institut für Hochenergiephysik, Vienna, Austria; 3 Universiteit Antwerpen, Antwerpen, Belgium; 4 Vrije Universiteit Brussel, Brussels, Belgium; 5 Université Libre de Bruxelles, Brussels, Belgium; 6 Ghent University, Ghent, Belgium; 7 Université Catholique de Louvain, Louvain-la-Neuve, Belgium; 8 Centro Brasileiro de Pesquisas Fisicas, Rio de Janeiro, Brazil; 9 Universidade do Estado do Rio de Janeiro, Rio de Janeiro, Brazil; 10 Universidade Estadual Paulista, Universidade Federal do ABC, São Paulo, Brazil; 11 Institute for Nuclear Research and Nuclear Energy, Bulgarian Academy of Sciences, Sofia, Bulgaria; 12 University of Sofia, Sofia, Bulgaria; 13 Instituto De Alta Investigación, Universidad de Tarapacá, Casilla 7 D, Arica, Chile; 14 Beihang University, Beijing, China; 15 Department of Physics, Tsinghua University, Beijing, China; 16 Institute of High Energy Physics, Beijing, China; 17 State Key Laboratory of Nuclear Physics and Technology, Peking University, Beijing, China; 18 Sun Yat-Sen University, Guangzhou, China; 19 University of Science and Technology of China, Hefei, China; 20 Institute of Modern Physics and Key Laboratory of Nuclear Physics and Ion-beam Application (MOE)-Fudan University, Shanghai, China; 21 Zhejiang University, Hangzhou, Zhejiang, China; 22 Universidad de Los Andes, Bogotá, Colombia; 23 Universidad de Antioquia, Medellin, Colombia; 24 University of Split, Faculty of Electrical Engineering, Mechanical Engineering and Naval Architecture, Split, Croatia; 25 University of Split, Faculty of Science, Split, Croatia; 26 Institute Rudjer Boskovic, Zagreb, Croatia; 27 University of Cyprus, Nicosia, Cyprus; 28 Charles University, Prague, Czech Republic; 29 Escuela Politecnica Nacional, Quito, Ecuador; 30 Universidad San Francisco de Quito, Quito, Ecuador; 31 Academy of Scientific Research and Technology of the Arab Republic of Egypt, Egyptian Network of High Energy Physics, Cairo, Egypt; 32 Center for High Energy Physics (CHEP-FU), Fayoum University, El-Fayoum, Egypt; 33 National Institute of Chemical Physics and Biophysics, Tallinn, Estonia; 34 Department of Physics, University of Helsinki, Helsinki, Finland; 35 Helsinki Institute of Physics, Helsinki, Finland; 36 Lappeenranta-Lahti University of Technology, Lappeenranta, Finland; 37 IRFU, CEA, Université Paris-Saclay, Gif-sur-Yvette, France; 38 Laboratoire Leprince-Ringuet, CNRS/IN2P3, Ecole Polytechnique, Institut Polytechnique de Paris, Palaiseau, France; 39 CNRS, IPHC UMR 7178, Université de Strasbourg, Strasbourg, France; 40 Institut de Physique des 2 Infinis de Lyon (IP2I ), Villeurbanne, France; 41 Georgian Technical University, Tbilisi, Georgia; 42 RWTH Aachen University, I. Physikalisches Institut, Aachen, Germany; 43 RWTH Aachen University, III. Physikalisches Institut A, Aachen, Germany; 44 RWTH Aachen University, III. Physikalisches Institut B, Aachen, Germany; 45 Deutsches Elektronen-Synchrotron, Hamburg, Germany; 46 University of Hamburg, Hamburg, Germany; 47 Karlsruher Institut fuer Technologie, Karlsruhe, Germany; 48 Institute of Nuclear and Particle Physics (INPP), NCSR Demokritos, Aghia Paraskevi, Greece; 49 National and Kapodistrian University of Athens, Athens, Greece; 50 National Technical University of Athens, Athens, Greece; 51 University of Ioánnina, Ioannina, Greece; 52 MTA-ELTE Lendület CMS Particle and Nuclear Physics Group, Eötvös Loránd University, Budapest, Hungary; 53 Wigner Research Centre for Physics, Budapest, Hungary; 54 Institute of Nuclear Research ATOMKI, Debrecen, Hungary; 55 Institute of Physics, University of Debrecen, Debrecen, Hungary; 56 Karoly Robert Campus, MATE Institute of Technology, Gyongyos, Hungary; 57 Panjab University, Chandigarh, India; 58 University of Delhi, Delhi, India; 59 Saha Institute of Nuclear Physics, HBNI, Kolkata, India; 60 Indian Institute of Technology Madras, Madras, India; 61 Bhabha Atomic Research Centre, Mumbai, India; 62 Tata Institute of Fundamental Research-A, Mumbai, India; 63 Tata Institute of Fundamental Research-B, Mumbai, India; 64 National Institute of Science Education and Research, An OCC of Homi Bhabha National Institute, Bhubaneswar, Odisha, India; 65 Indian Institute of Science Education and Research (IISER), Pune, India; 66 Isfahan University of Technology, Isfahan, Iran; 67 Institute for Research in Fundamental Sciences (IPM), Tehran, Iran; 68 University College Dublin, Dublin, Ireland; 69 INFN Sezione di Bari, Università di Bari, Politecnico di Bari, Bari, Italy; 70 INFN Sezione di Bologna, Università di Bologna, Bologna, Italy; 71 INFN Sezione di Catania, Università di Catania, Catania, Italy; 72 INFN Sezione di Firenze, Università di Firenze, Firenze, Italy; 73 INFN Laboratori Nazionali di Frascati, Frascati, Italy; 74 INFN Sezione di Genova, Università di Genova, Genoa, Italy; 75 INFN Sezione di Milano-Bicocca, Università di Milano-Bicocca, Milan, Italy; 76 INFN Sezione di Napoli, Università di Napoli ’Federico II’, Napoli, Italy; Università della Basilicata, Potenza, Italy;Università G. Marconi, Rome, Italy; 77 INFN Sezione di Padova, Università di Padova, Padova, Italy; Università di Trento, Trento, Italy; 78 INFN Sezione di Pavia, Università di Pavia, Pavia, Italy; 79 INFN Sezione di Perugia, Università di Perugia, Perugia, Italy; 80 INFN Sezione di Pisa, Università di Pisa, Scuola Normale Superiore di Pisa, Pisa, Italy; Università di Siena, Siena, Italy; 81 INFN Sezione di Roma, Sapienza Università di Roma, Rome, Italy; 82 INFN Sezione di Torino, Università di Torino, Torino, Italy; Università del Piemonte Orientale, Novara, Italy; 83 INFN Sezione di Trieste, Università di Trieste, Trieste, Italy; 84 Kyungpook National University, Daegu, Korea; 85 Chonnam National University, Institute for Universe and Elementary Particles, Kwangju, Korea; 86 Hanyang University, Seoul, Korea; 87 Korea University, Seoul, Korea; 88 Kyung Hee University, Department of Physics, Seoul, Korea; 89 Sejong University, Seoul, Korea; 90 Seoul National University, Seoul, Korea; 91 University of Seoul, Seoul, Korea; 92 Yonsei University, Department of Physics, Seoul, Korea; 93 Sungkyunkwan University, Suwon, Korea; 94 College of Engineering and Technology, American University of the Middle East (AUM), Dasman, Kuwait; 95 Riga Technical University, Riga, Latvia; 96 Vilnius University, Vilnius, Lithuania; 97 National Centre for Particle Physics, Universiti Malaya, Kuala Lumpur, Malaysia; 98 Universidad de Sonora (UNISON), Hermosillo, Mexico; 99 Centro de Investigacion y de Estudios Avanzados del IPN, Mexico City, Mexico; 100 Universidad Iberoamericana, Mexico City, Mexico; 101 Benemerita Universidad Autonoma de Puebla, Puebla, Mexico; 102 University of Montenegro, Podgorica, Montenegro; 103 National Centre for Physics, Quaid-I-Azam University, Islamabad, Pakistan; 104 AGH University of Science and Technology Faculty of Computer Science, Electronics and Telecommunications, Kraków, Poland; 105 National Centre for Nuclear Research, Swierk, Poland; 106 Institute of Experimental Physics, Faculty of Physics, University of Warsaw, Warsaw, Poland; 107 Laboratório de Instrumentação e Física Experimental de Partículas, Lisbon, Portugal; 108 VINCA Institute of Nuclear Sciences, University of Belgrade, Belgrade, Serbia; 109 Centro de Investigaciones Energéticas Medioambientales y Tecnológicas (CIEMAT), Madrid, Spain; 110 Universidad Autónoma de Madrid, Madrid, Spain; 111 Universidad de Oviedo, Instituto Universitario de Ciencias y Tecnologías Espaciales de Asturias (ICTEA), Oviedo, Spain; 112 Instituto de Física de Cantabria (IFCA), CSIC-Universidad de Cantabria, Santander, Spain; 113 University of Colombo, Colombo, Sri Lanka; 114 University of Ruhuna, Department of Physics, Matara, Sri Lanka; 115 CERN, European Organization for Nuclear Research, Geneva, Switzerland; 116 Paul Scherrer Institut, Villigen, Switzerland; 117 ETH Zurich-Institute for Particle Physics and Astrophysics (IPA), Zurich, Switzerland; 118 Universität Zürich, Zurich, Switzerland; 119 National Central University, Chung-Li, Taiwan; 120 National Taiwan University (NTU), Taipei, Taiwan; 121 Chulalongkorn University, Faculty of Science, Department of Physics, Bangkok, Thailand; 122 Çukurova University, Physics Department, Science and Art Faculty, Adana, Turkey; 123 Middle East Technical University, Physics Department, Ankara, Turkey; 124 Bogazici University, Istanbul, Turkey; 125 Istanbul Technical University, Istanbul, Turkey; 126 Istanbul University, Istanbul, Turkey; 127 Institute for Scintillation Materials of National Academy of Science of Ukraine, Kharkiv, Ukraine; 128 National Science Centre, Kharkiv Institute of Physics and Technology, Kharkiv, Ukraine; 129 University of Bristol, Bristol, United Kingdom; 130 Rutherford Appleton Laboratory, Didcot, UK; 131 Imperial College, London, UK; 132 Brunel University, Uxbridge, UK; 133 Baylor University, Waco, TX USA; 134 Catholic University of America, Washington, DC USA; 135 The University of Alabama, Tuscaloosa, AL USA; 136 Boston University, Boston, MA USA; 137 Brown University, Providence, RI USA; 138 University of California, Davis, Davis, CA USA; 139 University of California, Los Angeles, CA USA; 140 University of California, Riverside, Riverside, CA USA; 141 University of California, San Diego, La Jolla, CA USA; 142 Department of Physics, University of California, Santa Barbara, Santa Barbara, CA USA; 143 California Institute of Technology, Pasadena, CA USA; 144 Carnegie Mellon University, Pittsburgh, PA USA; 145 University of Colorado Boulder, Boulder, CO USA; 146 Cornell University, Ithaca, NY USA; 147 Fermi National Accelerator Laboratory, Batavia, IL USA; 148 University of Florida, Gainesville, FL USA; 149 Florida State University, Tallahassee, FL USA; 150 Florida Institute of Technology, Melbourne, FL USA; 151 University of Illinois at Chicago (UIC), Chicago, IL USA; 152 The University of Iowa, Iowa City, IA USA; 153 Johns Hopkins University, Baltimore, MD USA; 154 The University of Kansas, Lawrence, KS USA; 155 Kansas State University, Manhattan, KS USA; 156 Lawrence Livermore National Laboratory, Livermore, CA USA; 157 University of Maryland, College Park, MD USA; 158 Massachusetts Institute of Technology, Cambridge, MA USA; 159 University of Minnesota, Minneapolis, MN USA; 160 University of Mississippi, Oxford, MS USA; 161 University of Nebraska-Lincoln, Lincoln, NE USA; 162 State University of New York at Buffalo, Buffalo, NY USA; 163 Northeastern University, Boston, MA USA; 164 Northwestern University, Evanston, IL USA; 165 University of Notre Dame, Notre Dame, IN USA; 166 The Ohio State University, Columbus, OH USA; 167 Princeton University, Princeton, NJ USA; 168 University of Puerto Rico, Mayaguez, PR USA; 169 Purdue University, West Lafayette, IN USA; 170 Purdue University Northwest, Hammond, IN USA; 171 Rice University, Houston, TX USA; 172 University of Rochester, Rochester, NY USA; 173 The Rockefeller University, New York, NY USA; 174 Rutgers, The State University of New Jersey, Piscataway, NJ USA; 175 University of Tennessee, Knoxville, TN USA; 176 Texas A &M University, College Station, TX USA; 177 Texas Tech University, Lubbock, TX USA; 178 Vanderbilt University, Nashville, TN USA; 179 University of Virginia, Charlottesville, VA USA; 180 Wayne State University, Detroit, MI USA; 181 University of Wisconsin-Madison, Madison, WI USA; 182 Authors Affiliated with an Institute or an International Laboratory Covered by a Cooperation Agreement with CERN, Geneva, Switzerland; 183 Yerevan State University, Yerevan, Armenia; 184 TU Wien, Vienna, Austria; 185 Institute of Basic and Applied Sciences, Faculty of Engineering, Arab Academy for Science, Technology and Maritime Transport, Alexandria, Egypt; 186 Université Libre de Bruxelles, Brussels, Belgium; 187 Universidade Estadual de Campinas, Campinas, Brazil; 188 Federal University of Rio Grande do Sul, Porto Alegre, Brazil; 189 UFMS, Nova Andradina, Brazil; 190 University of Chinese Academy of Sciences, Beijing, China; 191 Nanjing Normal University, Nanjing, China; 192 The University of Iowa, Iowa City, IA USA; 193 University of Chinese Academy of Sciences, Beijing, China; 194 an Institute or an International Laboratory Covered by a Cooperation Agreement with CERN, Geneva, Switzerland; 195 Helwan University, Cairo, Egypt; 196 Zewail City of Science and Technology, Zewail, Egypt; 197 British University in Egypt, Cairo, Egypt; 198 Ain Shams University, Cairo, Egypt; 199 Purdue University, West Lafayette, IN USA; 200 Université de Haute Alsace, Mulhouse, France; 201 Department of Physics, Tsinghua University, Beijing, China; 202 The University of the State of Amazonas, Manaus, Brazil; 203 Erzincan Binali Yildirim University, Erzincan, Turkey; 204 University of Hamburg, Hamburg, Germany; 205 RWTH Aachen University, III. Physikalisches Institut A, Aachen, Germany; 206 Isfahan University of Technology, Isfahan, Iran; 207 Bergische University Wuppertal (BUW), Wuppertal, Germany; 208 Brandenburg University of Technology, Cottbus, Germany; 209 Forschungszentrum Jülich, Juelich, Germany; 210 CERN, European Organization for Nuclear Research, Geneva, Switzerland; 211 Physics Department, Faculty of Science, Assiut University, Assiut, Egypt; 212 Wigner Research Centre for Physics, Budapest, Hungary; 213 Institute of Physics, University of Debrecen, Debrecen, Hungary; 214 Institute of Nuclear Research ATOMKI, Debrecen, Hungary; 215 Universitatea Babes-Bolyai-Facultatea de Fizica, Cluj-Napoca, Romania; 216 Faculty of Informatics, University of Debrecen, Debrecen, Hungary; 217 Punjab Agricultural University, Ludhiana, India; 218 UPES-University of Petroleum and Energy Studies, Dehradun, India; 219 University of Visva-Bharati, Santiniketan, India; 220 University of Hyderabad, Hyderabad, India; 221 Indian Institute of Science (IISc), Bangalore, India; 222 Indian Institute of Technology (IIT), Mumbai, India; 223 IIT Bhubaneswar, Bhubaneswar, India; 224 Institute of Physics, Bhubaneswar, India; 225 Deutsches Elektronen-Synchrotron, Hamburg, Germany; 226 Department of Physics, Isfahan University of Technology, Isfahan, Iran; 227 Sharif University of Technology, Tehran, Iran; 228 Department of Physics, University of Science and Technology of Mazandaran, Behshahr, Iran; 229 Italian National Agency for New Technologies, Energy and Sustainable Economic Development, Bologna, Italy; 230 Centro Siciliano di Fisica Nucleare e di Struttura Della Materia, Catania, Italy; 231 Università degli Studi Guglielmo Marconi, Rome, Italy; 232 Scuola Superiore Meridionale, Università di Napoli ’Federico II’, Naples, Italy; 233 Fermi National Accelerator Laboratory, Batavia, IL USA; 234 Università di Napoli ’Federico II’, Naples, Italy; 235 Laboratori Nazionali di Legnaro dell’INFN, Legnaro, Italy; 236 Consiglio Nazionale delle Ricerche-Istituto Officina dei Materiali, Perugia, Italy; 237 Department of Applied Physics, Faculty of Science and Technology, Universiti Kebangsaan Malaysia, Bangi, Malaysia; 238 Consejo Nacional de Ciencia y Tecnología, Mexico City, Mexico; 239 IRFU, CEA, Université Paris-Saclay, Gif-sur-Yvette, France; 240 Faculty of Physics, University of Belgrade, Belgrade, Serbia; 241 Trincomalee Campus, Eastern University, Sri Lanka, Nilaveli, Sri Lanka; 242 INFN Sezione di Pavia, Università di Pavia, Pavia, Italy; 243 National and Kapodistrian University of Athens, Athens, Greece; 244 Ecole Polytechnique Fédérale Lausanne, Lausanne, Switzerland; 245 Universität Zürich, Zurich, Switzerland; 246 Stefan Meyer Institute for Subatomic Physics, Vienna, Austria; 247 Laboratoire d’Annecy-le-Vieux de Physique des Particules, IN2P3-CNRS, Annecy-le-Vieux, France; 248 Near East University, Research Center of Experimental Health Science, Mersin, Turkey; 249 Konya Technical University, Konya, Turkey; 250 Izmir Bakircay University, Izmir, Turkey; 251 Adiyaman University, Adiyaman, Turkey; 252 Istanbul Gedik University, Istanbul, Turkey; 253 Necmettin Erbakan University, Konya, Turkey; 254 Bozok Universitetesi Rektörlügü, Yozgat, Turkey; 255 Marmara University, Istanbul, Turkey; 256 Milli Savunma University, Istanbul, Turkey; 257 Kafkas University, Kars, Turkey; 258 Hacettepe University, Ankara, Turkey; 259 Istanbul University-Cerrahpasa, Faculty of Engineering, Istanbul, Turkey; 260 Ozyegin University, Istanbul, Turkey; 261 Vrije Universiteit Brussel, Brussel, Belgium; 262 School of Physics and Astronomy, University of Southampton, Southampton, UK; 263 University of Bristol, Bristol, UK; 264 IPPP Durham University, Durham, UK; 265 Faculty of Science, Monash University, Clayton, Australia; 266 Università di Torino, Turin, Italy; 267 Bethel University, St. Paul, MN USA; 268 Karamanoğlu Mehmetbey University, Karaman, Turkey; 269 California Institute of Technology, Pasadena, CA USA; 270 United States Naval Academy, Annapolis, MD USA; 271 Bingol University, Bingol, Turkey; 272 Georgian Technical University, Tbilisi, Georgia; 273 Sinop University, Sinop, Turkey; 274 Erciyes University, Kayseri, Turkey; 275 Texas A &M University at Qatar, Doha, Qatar; 276 Kyungpook National University, Daegu, Korea; 277 Another Institute or International Laboratory Covered by a Cooperation Agreement with CERN, Geneva, Switzerland; 278 Yerevan Physics Institute, Yerevan, Armenia; 279 Another Institute or International Laboratory Covered by a Cooperation Agreement with CERN, Geneva, Switzerland; 280 Imperial College, London, UK; 281 Institute of Nuclear Physics of the Uzbekistan Academy of Sciences, Tashkent, Uzbekistan; 282 CERN, 1211 Geneva 23, Switzerland

## Abstract

A search for decays to invisible particles of Higgs bosons produced in association with a top-antitop quark pair or a vector boson, which both decay to a fully hadronic final state, has been performed using proton-proton collision data collected at ${\sqrt{s}=13\,\text {Te}\hspace{-.08em}\text {V}}$ by the CMS experiment at the LHC, corresponding to an integrated luminosity of 138$\,\text {fb}^{-1}$. The 95% confidence level upper limit set on the branching fraction of the 125$\,\text {Ge}\hspace{-.08em}\text {V}$ Higgs boson to invisible particles, ${\mathcal {B}({\textrm{H}} \rightarrow \text {inv})}$, is 0.54 (0.39 expected), assuming standard model production cross sections. The results of this analysis are combined with previous ${\mathcal {B}({\textrm{H}} \rightarrow \text {inv})}$ searches carried out at ${\sqrt{s}=7}$, 8, and 13$\,\text {Te}\hspace{-.08em}\text {V}$ in complementary production modes. The combined upper limit at 95% confidence level on ${\mathcal {B}({\textrm{H}} \rightarrow \text {inv})}$ is 0.15 (0.08 expected).

## Introduction

The Higgs boson (H) [1–6] of mass 125$\,\text {Ge}\hspace{-.08em}\text {V}$ was discovered by the ATLAS and CMS Collaborations in 2012 [7–9]. Since then its properties, including its coupling to other standard model (SM) particles, have been extensively studied using proton-proton (${\textrm{pp}}$) collision data from the CERN LHC collected at ${\sqrt{s}=7}$, 8, and 13$\,\text {Te}\hspace{-.08em}\text {V}$ with the ATLAS [10] and CMS [11] detectors. Properties of the Higgs boson can be exploited to probe for signs of behaviour beyond the SM (BSM). In the SM, the decay of the Higgs boson to an invisible final state (${\textrm{H}} \rightarrow \text {inv}$) is only possible via ${\textrm{H}} \rightarrow {\textrm{Z}} {\textrm{Z}} ^* \rightarrow 4{{\upnu }}$, with a branching fraction of 0.1% [12]. Several BSM theories predict a larger branching fraction to invisible final states, ${\mathcal {B}({\textrm{H}} \rightarrow \text {inv})}$  [13–16], namely in Ref. [17] and references therein. For example, in a scenario where the Higgs boson connects the SM and dark matter (DM) sectors [18–23], ${\mathcal {B}({\textrm{H}} \rightarrow \text {inv})}$ is enhanced as the Higgs boson can decay to a pair of DM particles of mass $m_{\text {DM}}<m_{{\textrm{H}}}/2$.

Direct searches for ${\textrm{H}} \rightarrow \text {inv}$ have been performed by the ATLAS [24–29] and CMS [30–36] Collaborations using data collected during Run 1 (2011–2012) and Run 2 (2015–2018). These target channels in which the Higgs boson is produced via vector boson fusion (VBF), gluon-gluon fusion (ggH), and in association with either a vector boson (VH, where V stands for either a W or Z boson) or with a t $\overline{{{{\textrm{t}}}}}$ quark pair (${{\textrm{t}} {}{\overline{{{{\textrm{t}}}}}}} {\textrm{H}}$). The current most stringent constraint on ${\mathcal {B}({\textrm{H}} \rightarrow \text {inv})}$ set by the CMS experiment is via the VBF channel using Run 1 and Run 2 data, which reports a 95% confidence level ($\text {CL}$) upper limit of 0.18 (0.10 expected) [36].

**Fig. 1:**
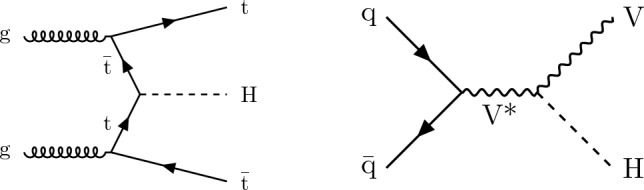
Representative LO Feynman diagrams for the SM Higgs boson production channels ${{\textrm{t}} {}{\overline{{{{\textrm{t}}}}}}} {\textrm{H}}$ and VH

In this paper, a search for an invisibly decaying Higgs boson, produced in association with a t $\overline{{{{\textrm{t}}}}}$ quark pair or a V boson, where the associated particles decay to a fully hadronic final state, is reported. Representative leading order (LO) Feynman diagrams for ${{\textrm{t}} {}{\overline{{{{\textrm{t}}}}}}} {\textrm{H}}$ and VH are presented in Fig. 1. The search in the VH channel looks only at topologies in which the presence of the V boson is inferred from well separated decay products, complementing the previous VH search with merged decay products arising from boosted V bosons [35]. The search uses LHC ${\textrm{pp}}$ collision data collected during the years 2016–2018, corresponding to a total integrated luminosity of 138$\,\text {fb}^{-1}$ at $\sqrt{s}=13\,\text {Te}\hspace{-.08em}\text {V}$. This is the first time that these final states have been used by the CMS experiment to search for the ${\textrm{H}} \rightarrow \text {inv}$ process using data from 2016–2018.

The missing transverse momentum, ${\vec p}_{\textrm{T}}^{\hspace{1.0pt}\text {miss}}$, is the transverse component of the negative vector sum of all reconstructed particle momenta in an event, and has a magnitude $p_{\textrm{T}} ^\text {miss}$. There are two main sources of background events resulting in ${\vec p}_{\textrm{T}}^{\hspace{1.0pt}\text {miss}}$ measurement. The first is events with invisible Z boson decays and visible jets (${\textrm{Z}} \rightarrow \text {inv}$). The second is referred to as the lost lepton background, $\ell _{\text {lost}}$, where $\ell$ stands for either an e or $\upmu$. This includes events from ${{\textrm{t}} {}{\overline{{{{\textrm{t}}}}}}} + \text {jets}$ and ${\textrm{W}} + \text {jets}$ processes where one or more leptons are misreconstructed, excluded by the phase space selection, or fall outside the detector acceptance. Control regions (CRs) enriched in these background sources, requiring either one lepton, one photon, or two same-flavour opposite-sign leptons, are used to constrain these backgrounds from data. The hadronic recoil is defined as the vectorial sum of the ${\vec p}_{\textrm{T}}^{\hspace{1.0pt}\text {miss}}$ and the $p_{\textrm{T}}$ of any selected charged lepton(s) or photon in an event, and its magnitude is used as the discriminating variable to separate the ${\textrm{H}} \rightarrow \text {inv}$ signal from backgrounds. The 95% $\text {CL}$ upper limit on ${\mathcal {B}({\textrm{H}} \rightarrow \text {inv})}$ is extracted from a fit to the hadronic recoil distribution of selected events, performed across the signal regions (SRs) and CRs. In the SRs, the hadronic recoil is equivalent to the $p_{\textrm{T}} ^\text {miss}$, while in the CRs it effectively measures the $p_{\textrm{T}}$ of the V boson or photon. The exclusion of leptons and photons ensures good correspondence between SRs and CRs.

This paper is organised as follows: Section 2 is a brief description of the CMS detector. The simulated samples used in this analysis are summarised in Sect. 3. Section 4 describes the event reconstruction and object definitions used in this analysis, while the event selection and event categorisation are detailed in Sect. 5. The data CRs used for estimating the SM backgrounds are introduced in Sect. 6. Section 7 describes the statistical procedure used to constrain the backgrounds and extract the signal. The results of the search are presented in Sect. 8. The results of combining this search with other CMS searches for invisibly decaying Higgs bosons are described in Sect. 9, and the results are summarised in Sect. 10.

## The CMS detector

The CMS apparatus is a multipurpose, nearly hermetic detector, designed to trigger on [37, 38] and identify electrons, muons, photons, and (charged and neutral) hadrons [39–41]. The central feature of the CMS apparatus is a superconducting solenoid, of $6~\textrm{m}$ internal diameter. Within the field volume are the silicon pixel and strip tracker, the crystal electromagnetic calorimeter (ECAL), and the brass-scintillator hadron calorimeter (HCAL). Muons are measured in gas-ionisation chambers embedded in the steel flux-return yoke of the magnet. Besides the barrel and endcap detectors, CMS has extensive forward calorimetry, performed on high $\eta$ objects in the HCAL forward calorimeter, which is located $11.2~\textrm{m}$ from the interaction region along the beam axis. A global “particle-flow” (PF) algorithm [42] aims to reconstruct all individual particles in an event, combining information provided by all subdetectors. The reconstructed particles are used to build $\uptau$ leptons, jets, and $p_{\textrm{T}} ^\text {miss}$  [43–45].

The first level of the CMS trigger system, composed of custom hardware processors, uses information from the calorimeters and muon detectors to select the most interesting events, at a rate of roughly $100~\textrm{kHz}$. The high-level trigger (HLT) processor farm performs event reconstruction similar to that of the full CMS reconstruction, but optimised for speed. This decreases the event rate from around $100\text {\,kHz}$ to around $1\text {\,kHz}$, before data storage [38].

The procedures for calculating the integrated luminosity recorded by the CMS detector for each data-taking year are documented in Refs. [46–48] for 2016–2018, respectively.

A more detailed description of the CMS experiment can be found in Ref. [49].

## Simulated samples

Monte Carlo (MC) simulated events are used to model signal and background contributions in all analysis regions, except for quantum chromodynamics (QCD) multijet production processes, which are estimated from data using a dedicated control sample and simulation-based transfer factors. The method for estimating QCD multijet production is detailed in Sect. 6.2. In all cases, MC samples are produced using either powheg version 1.0 or higher [50] or MadGraph 5_amc@nlo version 2.4.2 or higher [51] matrix element (ME) generators. The ME is encoded with the maximum amount of information available for a hard scattering event. The parton-level simulation provided by the ME generators is interfaced with pythia version 8 [52] to model the shower and hadronisation of partons in the initial and final states, along with the underlying event description, using the tune CUETP8M1 (CP5) when simulating events for the 2016 (2017 and 2018) data-taking periods [53]. The propagation of all final state particles through the CMS detector is simulated using the Geant4  [54] toolkit. Samples for 2016 make use of the NNPDF3.0 LO or next-to-LO (NLO) parton distribution functions (PDFs) [55], whereas samples for the years 2017 and 2018 use the NNPDF3.1 next-to-NLO (NNLO) PDFs.

Processes featuring ${\textrm{H}} \rightarrow \text {inv}$ occurring in ${{\textrm{t}} {}{\overline{{{{\textrm{t}}}}}}} {\textrm{H}}$, VH, VBF, and ggH channels are modelled by powheg version 2.0 [56–59] at NLO in QCD. These samples require the SM Higgs boson to decay to four neutrinos (${\textrm{H}} \rightarrow {\textrm{Z}} {\textrm{Z}} ^* \rightarrow 4{{\upnu }}$) resulting in ${\mathcal {B}({\textrm{H}} \rightarrow \text {inv})}$ $=1$. The cross sections are appropriately normalised to the corresponding SM predictions computed at NLO (${{\textrm{t}} {}{\overline{{{{\textrm{t}}}}}}} {\textrm{H}}$), NNLO (VH, VBF), and next-to-NNLO (ggH) accuracy in QCD, and to NLO accuracy in electroweak (EW) corrections [60]. Background ZH processes with the Higgs boson decaying to ${\textrm{b}} \overline{{\textrm{b}}}$ and the associated Z boson decaying to ${\textrm{b}} \overline{{\textrm{b}}}$, ${\ell \ell }$, and ${\textrm{q}} {\overline{{{{\textrm{q}}}}}}$ (where q represents a light or charm quark) are generated at NLO using MadGraph 5_amc@nlo with the FxFx [61] matching scheme for 2016 samples, and with powheg version 2.0 [62] for 2017 and 2018 samples.

The ${\textrm{V}} + \text {jets}$ processes are generated at LO in QCD using MadGraph 5_amc@nlo with up to four partons in the final state using the MLM [63] matching scheme between hard scatters and parton showers. These processes are generated in bins of hadronic transverse energy, $H_{\textrm{T}}$, which is the magnitude of the ${\vec p}_{\textrm{T}}$ sum of all jets reconstructed at generator level. The LO simulation of ${\textrm{V}} + \text {jets}$ processes is corrected to account for missing higher-order diagrams with K-factors derived from MadGraph 5_amc@nlo-generated NLO QCD ${\textrm{V}} + \text {jets}$ processes with up to two partons. These K-factors are extracted as a function of boson $p_{\textrm{T}}$ and the $p_{\textrm{T}}$ of the leading jet in the event. These K-factors are extracted as a function of boson $p_{\textrm{T}}$ and the $p_{\textrm{T}}$ of the leading jet in the event, and typically vary between 0.5 and 1.5 depending on the boson $p_{\textrm{T}}$.

The ${\upgamma } + \text {jets}$ processes are generated at NLO in QCD with MadGraph 5_amc@nlo, using a binning based on the $p_{\textrm{T}}$ of the photon. The binning scheme for this sample is defined at the ME level to increase the statistical precision in the phase space regions probed by this analysis.

Processes including *t*-channel single t quarks, and t $\overline{{{{\textrm{t}}}}}$ pairs with up to two additional partons in ME computation are generated at NLO with powheg version 2.0 [64, 65]. Single t quarks produced in the *s* channel are modelled using MadGraph 5_amc@nlo, and also in the *tW* channel using powheg version 1.0 [66]. The t quark $p_{\textrm{T}}$ spectrum in t $\overline{{{{\textrm{t}}}}}$ processes is corrected to match the spectrum obtained from the NNLO QCD $+$ NLO EW simulation, following Ref. [67]. Rare ${{{\textrm{t}} {}{\overline{{{{\textrm{t}}}}}}} {\textrm{X}}}$ $+$ jets backgrounds cover processes where t $\overline{{{{\textrm{t}}}}}$ is produced in association with a boson X ($\upgamma$, V, or a visibly decaying H), generated at NLO. The ${{{\textrm{t}} {}{\overline{{{{\textrm{t}}}}}}} {\upgamma }}$ $+$ jets, ${{{\textrm{t}} {}{\overline{{{{\textrm{t}}}}}}} {\textrm{W}}}$ $+$ jets, and ${{{\textrm{t}} {}{\overline{{{{\textrm{t}}}}}}} {\textrm{Z}}}$ $+$ jets samples are generated using MadGraph 5_amc@nlo, with subsequent decays generated using MadSpin [68] to account for spin correlations in the former two cases. The ${{\textrm{t}} {}{\overline{{{{\textrm{t}}}}}}} {\textrm{H}}$  $+$ jets sample, where the H decays to visible states, is generated using powheg.

Diboson ${\textrm{ZZ}}$ and ${\textrm{WZ}}$ production processes are generated at LO using pythia, while the ${\textrm{WW}}$ process is simulated at NLO in QCD using the powheg version 2.0 [69]. The QCD multijet samples are generated at LO using MadGraph 5_amc@nlo in exclusive ranges of $H_{\textrm{T}}$ in order to increase the statistical precision in the phase-space probed by this analysis.

## Event reconstruction

During LHC runs, each beam crossing results in several ${\textrm{pp}}$ collisions in the detector. Additional ${\textrm{pp}}$ interactions within the same or nearby bunch crossing, known as pileup, make PF object reconstruction more challenging. The reduction of the effect of pileup relies on mitigation techniques [70] that filter energy deposits associated with pileup vertices and remove objects not associated with the primary interaction vertex (PV). The PV is the vertex associated with the hardest scattering in the event, according to tracking information, as described in Ref. [71]. All simulated samples from Section 3 are reweighted to match the pileup distribution observed in data. In the SR, the final state is required to contain jets, a sizeable hadronic recoil, and no isolated leptons or photons. Candidate leptons and photons are selected with ${p_{\textrm{T}} >10\,\text {Ge}\hspace{-.08em}\text {V}}$ and pseudorapidity ${|\eta | < 2.4}$ for muons [40], ${p_{\textrm{T}} >10\,\text {Ge}\hspace{-.08em}\text {V}}$ and either ${|\eta | < 1.44}$ or ${1.57< |\eta | < 2.5}$ for electrons [39], ${p_{\textrm{T}} >20\,\text {Ge}\hspace{-.08em}\text {V}}$ and ${|\eta | < 2.3}$ for hadronically decaying tau leptons [43], and ${p_{\textrm{T}} >15\,\text {Ge}\hspace{-.08em}\text {V}}$ and either ${|\eta | < 1.44}$ or ${1.57< |\eta | < 2.5}$ for photons [39]. These selection criteria are optimised to reject background contributions, mainly from QCD processes. Other selection criteria depend on the isolation of the lepton or photon from hadronic interactions in the detector within a cone of small (tight isolation) or large (loose isolation) radius. Loose identification and isolation criteria are used to veto candidate events in the SR that contain leptons or photons. The veto efficiencies are > 99, $\simeq$ 95, and $\simeq$ 90% for loose muons, electrons, and photons, respectively. The SR background contributions are estimated using ${\upmu } + \text {jets}$, ${\textrm{e}} + \text {jets}$, ${\upmu \upmu }+$ jets, ee $+$ jets, and ${\upgamma } + \text {jets}$ CRs. Tight and loose identification and isolation criteria are used to select and count muons, electrons, and photons in the CRs, enhancing the purity at little expense to the efficiency. These achieve typical selection efficiencies of $\simeq$ 95, 70, and 70 ($\simeq$ 98, 95 and 90)%, for tight (loose) muons, electrons, and photons, respectively.

Jets are reconstructed by clustering all PF candidates originating from the PV with the anti-$k_{\textrm{T}}$ jet clustering algorithm [72, 73], using a distance parameter ${R=0.4}$ (AK4). Jet momentum is determined as the vectorial sum of all particle momenta in the jet, and is found from simulation to be, on average, within 5–10% of the true momentum over the whole $p_{\textrm{T}}$ spectrum and detector acceptance. Charged-hadron subtraction [44] is then applied to remove charged particles from pileup vertices [74]. To ensure the measured jet energy matches that of the particle level jets, jet energy corrections (JEC) derived from simulation as functions of $p_{\textrm{T}}$ and $\eta$ are applied. Further corrections are applied due to residual discrepancies in the jet energy scale (JES) between data and simulated samples [44]. Additionally, each jet must pass selection criteria to remove jets adversely affected by instrumentation or reconstruction failure. The jet energy resolution (JER) in simulated samples is smeared to match the measured resolution, which is typically 15–20% at 30$\,\text {Ge}\hspace{-.08em}\text {V}$, 10% at 100$\,\text {Ge}\hspace{-.08em}\text {V}$, and 5% at 1$\,\text {Te}\hspace{-.08em}\text {V}$  [44]. The AK4 jets are required to have ${p_{\textrm{T}} >30\,\text {Ge}\hspace{-.08em}\text {V}}$ and ${|\eta |<5.0}$, and those with loose leptons and photons located within a cone of ${\Delta R<0.4}$ of the jet direction are removed.

The AK4 jets that originate from the hadronisation of a bottom quark (b-tagged jets) are identified using the DeepCSV algorithm, which correctly identifies b jets with ${p_{\textrm{T}} >20\,\text {Ge}\hspace{-.08em}\text {V}}$ with a probability of 80% and has a charm or light jet mistag probability of 10% [75]. Simulated events containing b jets are corrected to be in agreement with the data by deriving corrections from data control samples that contain b jets.

Pileup effects are mitigated at the reconstructed particle level using the pileup per particle identification algorithm (PUPPI) [76, 77] by defining a local shape variable that can discriminate between particles originating from the PV and from pileup. Charged particles originating from pileup are discarded. For neutral particles, a local shape variable is computed based on the information from charged particles in their vicinity that originate from the PV within the tracker acceptance, and information from both charged and neutral particles outside this acceptance. The momenta of neutral particles are then rescaled based on the probability that they originated from the PV as deduced from the local shape variable [76].

When a high $p_{\textrm{T}}$ t quark or V boson decays hadronically, a large set of collimated particles cross the detector. These can be clustered within a single jet of radius $R=0.8$ (AK8) using the anti-$k_{\textrm{T}}$ algorithm. In order to reduce pileup effects, PUPPI PF candidates are used to seed the AK8 jet finder. The main feature that distinguishes hadronically decaying t quarks or V bosons from the quark or gluon fragmentation is the jet mass. To improve the resolution, the modified mass-drop tagger algorithm [78–80] (also known as the soft-drop algorithm, SD) with the angular exponent ${\beta =0}$, soft cutoff threshold ${z_{\text {cut}}<0.1}$, and characteristic radius ${R_{0}=0.8}$ [81] is applied to each AK8 jet to remove soft and wide-angle radiation. In addition, a deep neural network (DNN) classifier called the DeepAK8 [82] algorithm is employed by assigning a set of numerical scores to each reconstructed AK8 jet corresponding to the probabilities that it originates from particular final states of V boson decays, for example ${{\textrm{Z}} \rightarrow \textrm{b}\bar{\textrm{b}}}$, ${{\textrm{Z}} \rightarrow {\textrm{q}} {\overline{{{{\textrm{q}}}}}}}$, ${{\textrm{W}} \rightarrow {\textrm{cs}}}$, rather than from QCD multijet processes. For this analysis, reconstructed AK8 jets originating from t quarks (W bosons) are selected by requiring ${p_{\textrm{T}} >400~(200)\,\text {Ge}\hspace{-.08em}\text {V}}$, SD mass $m_{\text {SD}}$ between 120 and 210 (65 and 120)$\,\text {Ge}\hspace{-.08em}\text {V}$, and a DeepAK8 probability score for t quarks (W bosons) larger than between 72.5 and 83.4 (91.8 and 92.5)% depending on the year of data-taking. The resulting t quark (W boson) tagging efficiency at the ${p_{\textrm{T}} >400~(200)\,\text {Ge}\hspace{-.08em}\text {V}}$ threshold limit is estimated from simulation as 28 (25)% with a 1% mistag rate from QCD jets. Simulated events containing AK8 jets are corrected to agree with the data using data-derived correction factors, and dedicated JEC are also applied [82].

The calculation of energy sums such as the hadronic recoil, ${\vec p}_{\textrm{T}}^{\hspace{1.0pt}\text {miss}}$, and $\vec {H}_{\text {T}}^{\text {miss}}$, which is the negative ${\vec p}_{\textrm{T}}$ sum of jets reconstructed at the HLT level with a $p_{\textrm{T}} ^\text {miss}$ threshold of 20$\,\text {Ge}\hspace{-.08em}\text {V}$ applied, are based on AK4 jets, therefore JEC are propagated through the use of the ${\vec p}_{\textrm{T}}$-corrected jets.

## Event selection and categorisation

In this analysis the signal is extracted from a combined fit to the hadronic recoil distribution of events in SRs and CRs as defined for the ${{\textrm{t}} {}{\overline{{{{\textrm{t}}}}}}} {\textrm{H}}$ and VH categories. The CRs are used to estimate the contributions of different SM processes in each SR. Where possible, the CRs have kinematic requirements identical to the SR, and leptons or a photon are used in the CR definition, but otherwise ignored in the calculation of event observables. The ${\textrm{e}} + \text {jets}$ and ${\upmu } + \text {jets}$ CRs, enriched in ${\textrm{W}} + \text {jets}$ and t quark background processes, are used to derive corrections to $\ell _{\text {lost}}$ contributions predicted by simulation. The ee $+$ jets, ${\upmu \upmu }\,+$ jets, and, in the case of the VH category, ${\upgamma } + \text {jets}$ CR samples are used to derive corrections to the expected contribution from ${\textrm{Z}} + \text {jets}$ production, where the Z boson decays to a pair of neutrinos. A QCD multijet enriched CR (hadronic sideband) is also used to estimate hadronic backgrounds in the SR.

### Trigger requirements

Events of interest are collected via a suite of triggers that are applied to variables calculated using PF candidates reconstructed at the level of the HLT. The trigger requirements vary amongst analysis regions and data-taking periods. Events in the SR, hadronic sideband, and muon CRs are collected using HLT selection criteria on $p_{\textrm{T}} ^\text {miss}$ and the missing $H_{\textrm{T}}$, $H_\textrm{T}^\text {miss}$, which is the magnitude of $\vec {H}_{\text {T}}^{\text {miss}}$. Muons are not considered in the calculation of PF $p_{\textrm{T}} ^\text {miss}$ and PF $H_\textrm{T}^\text {miss}$ to allow the same trigger to be used in the SR and the muon CRs, with a typical efficiency of >90% for $p_{\textrm{T}} ^\text {miss}$ $>250\,\text {Ge}\hspace{-.08em}\text {V}$. The use of the combined $p_{\textrm{T}} ^\text {miss}$ and $H_\textrm{T}^\text {miss}$ triggers in the muon CRs instead of single-muon triggers corresponds more closely to the selection in the SR and minimises selection biases. Trigger thresholds increase with time due to the increase in instantaneous luminosity during Run 2. In 2016, the $p_{\textrm{T}} ^\text {miss}$ and $H_\textrm{T}^\text {miss}$ thresholds vary between 90 and 120$\,\text {Ge}\hspace{-.08em}\text {V}$. In 2017 and 2018, these thresholds are 120$\,\text {Ge}\hspace{-.08em}\text {V}$. During data-taking in 2017, additional corrections were applied to account for the effect of ECAL endcap noise at high $|\eta |$ on PF $p_{\textrm{T}} ^\text {miss}$ measurements. Additionally, for 2016 and 2017 data-taking periods, there was an inefficiency arising from a gradual shift in the timing of the ECAL trigger inputs in the region ${|\eta |>2.0}$ [37]. This resulted in events containing an electron or photon (jet) with ${p_{\textrm{T}} >50~(100)\,\text {Ge}\hspace{-.08em}\text {V}}$ having an efficiency loss of up to 20%, depending on $p_{\textrm{T}}$ and $\eta$. Correction factors for this trigger inefficiency are obtained from 2016 and 2017 data and applied to simulation samples as a function of $\eta$.

Events in the ${\textrm{e}} + \text {jets}$ and ee $+$ jets CRs from the 2016, 2017, and 2018 data sets are required to pass a tight (loose) single-electron trigger with $p_{\textrm{T}}$ thresholds of 27, 35, and 32 (105, 115, and 115)$\,\text {Ge}\hspace{-.08em}\text {V}$, respectively. The low-threshold single-electron triggers require the electron candidate to pass a tight isolation condition, while the high-threshold trigger imposes a looser selection on the isolation to improve the efficiency at high $p_{\textrm{T}}$. Photon events are required to pass a single-photon trigger with a $p_{\textrm{T}}$ threshold of 175 (200)$\,\text {Ge}\hspace{-.08em}\text {V}$ without any isolation condition for the 2016 (2017 and 2018) data sets. Simulated electron or photon events are accepted if they pass exactly one of the above trigger requirements, and the efficiency of this selection is corrected with data-derived efficiency correction factors.

### Offline selection

In order to select events with a large amount of jet activity and sizeable hadronic recoil, a further offline selection is applied to all regions. To improve the purity of the signal, large missing energy is desirable, therefore events require the hadronic recoil, $H_\textrm{T}^\text {miss}$, and $H_{\textrm{T}}$ to be greater than 200$\,\text {Ge}\hspace{-.08em}\text {V}$. Furthermore, the largest $p_{\textrm{T}}$ of an AK4 jet in an event, $\vec {p}^{~\text {j}}_\textrm{T,1}$, is required to be greater than 80$\,\text {Ge}\hspace{-.08em}\text {V}$. To ensure consistency amongst different estimators of the hadronic recoil, the recoil as calculated from PF candidates in $p_{\textrm{T}} ^\text {miss}$ and from PF jets in $H_\textrm{T}^\text {miss}$ must satisfy ${H_\textrm{T}^\text {miss}/\text {recoil}<1.2}$ and azimuthal separation ${|\Delta \phi (\overrightarrow{\text {recoil}}, \vec {H}_{\text {T}}^{\text {miss}}) |<0.5}$. To further improve the quality of events, a selection is made on $p_{\textrm{T},\text {track}}^{\text {miss}}$, which is equivalent to $p_{\textrm{T}} ^\text {miss}$ but calculated using only charged PF particles, and therefore is expected to be well-aligned with the hadronic recoil direction. Requirements of ${p_{\textrm{T},\text {track}}^{\text {miss}} >60\,\text {Ge}\hspace{-.08em}\text {V}}$ and azimuthal separation ${|\Delta \phi (\overrightarrow{\text {recoil}}, \vec {p}_{\textrm{T},\text {track}}^{\text {miss}}) |<1}$ are applied in the SR and hadronic sideband. The kinematic selection for all regions is optimised according to the Asimov significance between signal (S) and background (B) yields assuming a background systematic uncertainty $\Delta \text {B}$ of 5% or 10% [83]. The peaks of the distribution for a given variable corresponds to its selection threshold.

In order to facilitate the combination of this analysis with the results from other ${\textrm{H}} \rightarrow \text {inv}$ searches, additional selections are introduced to reduce the potential event overlap. A veto is implemented to ensure orthogonality with the VBF phase space, through a veto on events with leading (subleading) AK4 jets with ${|\eta _{1} |~(|\eta _{2} |)>2.4}$, and an inversion of the kinematic selection employed by the VBF ${\textrm{H}} \rightarrow \text {inv}$ analysis [36]. This removes events containing two AK4 jets with $\vec {p}^{~\text {j}}_\textrm{T,1} >80\,\text {Ge}\hspace{-.08em}\text {V}$ and the subleading jet $p_{\textrm{T}}$, $\vec {p}^{~\text {j}}_\textrm{T,2}$, to be greater than 40$\,\text {Ge}\hspace{-.08em}\text {V}$, where the jets are from opposite detector hemispheres (${\eta _{1}\eta _{2} < 0}$), have a large $m_{\text {jj}}$ (>200$\,\text {Ge}\hspace{-.08em}\text {V}$), small azimuthal separation (${\Delta \phi _{\text {jj}} < 1.5}$), and a large $\eta$ gap (${|\eta _{m_{\text {jj}}} | > 1.0}$). Moreover, orthogonality to leptonic ${{\textrm{t}} {}{\overline{{{{\textrm{t}}}}}}} {\textrm{H}}$ decays is ensured in the single-lepton CRs by requiring the transverse mass of the combined single-lepton and hadronic recoil system, defined as

1 $$\begin{aligned} m_\textrm{T}^{\ell } = \sqrt{2 p^{\ell }_{\textrm{T}} (\text {recoil}) [1 - \cos {(\phi (\vec {p}^{\ell }_{\textrm{T}}) - \phi (\overrightarrow{\text {recoil}}))}]}, \end{aligned}$$

to be lower than 110$\,\text {Ge}\hspace{-.08em}\text {V}$. Orthogonality between leptonic ${{\textrm{t}} {}{\overline{{{{\textrm{t}}}}}}} {\textrm{H}}$ decays in the dilepton CRs is ensured by requiring the invariant mass of the charged lepton pair, $m_{\ell \ell }$, to be lower than 120$\,\text {Ge}\hspace{-.08em}\text {V}$ in these CRs. Selecting on the invariant masses of lepton pairs also suppresses the ${{\textrm{t}} {}{\overline{{{{\textrm{t}}}}}}} {\textrm{H}}$ signal contamination in the CRs. Overlap between the ggH/boosted VH ${\textrm{H}} \rightarrow \text {inv}$ analysis and the resolved VH category of this analysis is rendered negligible by explicitly removing events from the low-purity boosted VH category defined in Ref. [35] if they contain exactly two AK4 jets with an invariant mass, $m_{\text {jj}}$, forming a dijet candidate with $65< m_{\text {jj}} < 120\,\text {Ge}\hspace{-.08em}\text {V}$. No corresponding selection is necessary for the resolved VH category as a result, while there is negligible change to the sensitivity of the boosted VH category.

During significant periods of data-taking in 2018, the HCAL portion corresponding to the region $-1.57<\phi <-0.87$, $-3.0<\eta <-1.39$ was not functional. Events from 2018 with $-1.8<\phi (\overrightarrow{\text {recoil}})<-0.6$ are vetoed if they contain jets within the affected region, which removes $\approx$65% of the total data from the affected region. To ensure good correspondence between data and simulation, the simulation is reweighted to account for the efficiency loss. A summary of the offline requirements are provided in Table 1.

**Table 1:** Offline selection applied to all categories and regions in this analysis to improve signal purity and reduce overlap with the phase space of other ${\textrm{H}} \rightarrow \text {inv}$ searches

Variable	Selection	Purpose
recoil	$>200\,\text {Ge}\hspace{-.08em}\text {V}$	Signal purity
$H_\textrm{T}^\text {miss}$	$>200\,\text {Ge}\hspace{-.08em}\text {V}$	
$\vec {p}^{~\text {j}}_\textrm{T,1}$	$>80\,\text {Ge}\hspace{-.08em}\text {V}$	
$H_\textrm{T}^\text {miss}/\text {recoil}$	$<1.2$	Event quality
$$|\Delta \phi (\overrightarrow{\text {recoil}}, \vec {H}_{\text {T}}^{\text {miss}}) |$$	$$<0.5 $$	
$|\eta _1 |$, $|\eta _2 |$	$<2.4$	Analysis orthogonalisation
VBF signal	Veto (inversion on signal selection)	
$m_{T}^{\ell }$	$<110\,\text {Ge}\hspace{-.08em}\text {V}$	
$m_{\ell \ell }$	$<120\,\text {Ge}\hspace{-.08em}\text {V}$	

### Signal regions

The search focuses on three types of hadronic final states: those with boosted t quarks and/or boosted W bosons reconstructed with dedicated merged jet algorithms; those with one or more b jets and no boosted t quark or W boson, targetting the bulk of hadronic ${{\textrm{t}} {}{\overline{{{{\textrm{t}}}}}}} {\textrm{H}}$ events; and those with two resolved jets with the $m_{\text {jj}}$ compatible with that of a W or Z boson. The latter complements the boosted VH channel analysed in Ref. [35].

Events are categorised into boosted and resolved ${{\textrm{t}} {}{\overline{{{{\textrm{t}}}}}}} {\textrm{H}}$, and resolved VH topologies. The ${{\textrm{t}} {}{\overline{{{{\textrm{t}}}}}}} {\textrm{H}}$ category requires that at least five AK4 jets and one b jet are present. The boosted ${{\textrm{t}} {}{\overline{{{{\textrm{t}}}}}}} {\textrm{H}}$ topology requires that at least one AK8 jet is reconstructed and either t- or W-tagged, and is subcategorised by the AK8 jet and b jet multiplicities. Events without such t- or W-tagged jets are categorised as belonging to a resolved ${{\textrm{t}} {}{\overline{{{{\textrm{t}}}}}}} {\textrm{H}}$ topology, with further selections on the leading AK4 jet (leading or subleading b jet) ${\vec p}_{\textrm{T}}$ and the hadronic recoil, $|\Delta \phi (\overrightarrow{\text {recoil}}, \vec {p}^{~\text {j}}_\textrm{T,1}) |$ ($|\Delta \phi (\overrightarrow{\text {recoil}}, \vec {p}^{~\text {b}}_\textrm{T,1}) |$ or $|\Delta \phi (\overrightarrow{\text {recoil}}, \vec {p}^{~\text {b}}_\textrm{T,2}) |$) applied to discriminate between ${{\textrm{t}} {}{\overline{{{{\textrm{t}}}}}}} {\textrm{H}}$ and ${{\textrm{t}} {}{\overline{{{{\textrm{t}}}}}}} + \text {jets}$ processes. Finally, the remaining events are allocated to the resolved VH topology category if they have exactly two AK4 jets with $m_{\text {jj}}$ between 65 and 120$\,\text {Ge}\hspace{-.08em}\text {V}$, compatible with a W or Z boson decay. The resolved VH subcategories are separated according to the b jet multiplicity. Subcategories are also defined based on $\vec {p}^{~\text {j}}_\textrm{T,2}$ to suppress QCD multijet background. The subcategory definitions are summarised in Table 2. The intended outcome of this categorisation is a set of event samples with high purity for a given production mode, and minimal background contamination or signal cross-contamination.

**Table 2:** Categorisation of the ${{\textrm{t}} {}{\overline{{{{\textrm{t}}}}}}} {\textrm{H}}$ and VH production modes in the analysis. No additional selections are applied to the boosted ${{\textrm{t}} {}{\overline{{{{\textrm{t}}}}}}} {\textrm{H}}$ subcategories

Category	Subcategory	$n_\text {j}$	$n_{{\textrm{b}}}$	$n_{{\textrm{t}}}$	$n_{{\textrm{W}}}$	$\vec {p}^{~\text {j}}_\textrm{T,2}$ ($\text {Ge}\hspace{-.08em}\text {V}$)	Other
	2Boosted1b	$\ge 5$	1	2			
	2Boosted2b	$\ge 5$	$\ge 2$	2			
Boosted $${{\textrm{t}} {}{\overline{{{{\textrm{t}}}}}}} {\textrm{H}} $$	1t1b	$$\ge 5$$	1	1	0	$$>80 $$	–
	1t2b	$\ge 5$	$\ge 2$	1	0		
	1W1b	$\ge 5$	1	0	1		
	1W2b	$\ge 5$	$\ge 2$	0	1		
	5j1b	5	1	0	0		$|\Delta \phi (\overrightarrow{\text {recoil}}, \vec {p}^{~\text {b}}_\textrm{T,1}) | > 1.0$,
Resolved $${{\textrm{t}} {}{\overline{{{{\textrm{t}}}}}}} {\textrm{H}} $$	6j1b	$$\ge 6$$	1	0	0	$$>80 $$	$$|\Delta \phi (\overrightarrow{\text {recoil}}, \vec {p}^{~\text {j}}_\textrm{T,1}) | > \pi /2$$
	5j2b	5	$\ge 2$	0	0		$|\Delta \phi (\overrightarrow{\text {recoil}}, \vec {p}^{~\text {b}}_\textrm{T,1}) | > 1.0$,
	6j2b	$\ge 6$	$\ge 2$	0	0		$|\Delta \phi (\overrightarrow{\text {recoil}}, \vec {p}^{~\text {b}}_\textrm{T,2}) | > \pi /2$
	2j0b	2	0	0	0		
VH	2j1b	2	1	0	0	$> 30$	$65< m_\text {jj} < 120\,\text {Ge}\hspace{-.08em}\text {V}$
	2j2b	2	2	0	0		

A requirement on $|\Delta \phi _\text {min}({{\vec p}_{\textrm{T}}^{\hspace{1.0pt}\text {miss}}, \vec {p}_\textrm{T,1234})} |$, defined as the minimum azimuthal separation between the hadronic recoil and the momentum direction of any of the four highest $p_{\textrm{T}}$ jets, of ${>}0.5$ is applied to suppress QCD multijet events where the hadronic recoil is aligned with a jet. A parameter $\tilde{\omega }_{\text {min}}$ is designed to suppress events where missing energy is the result of a jet $p_{\textrm{T}}$ mismeasurement, and is especially effective in categories with no b jets. For the *i*th jet in the event, $\omega _{i}$ is defined as $\arctan {(H_{\textrm{T}, \text {min}}^{\text {miss}} / p_{\textrm{T},i})}$, where $p_{\textrm{T},i}$ is the $p_{\textrm{T}}$ of jet *i*, and $H_{\textrm{T}, \text {min}}^{\text {miss}}$ is the minimum value of $H_\textrm{T}^\text {miss}$ that can be obtained by changing the value of $p_{\textrm{T},i}$. The value of $\omega _{i}$ minimised over *i* is $\tilde{\omega }_{\text {min}}$. A detailed derivation of this variable is given in Ref. [84]. QCD multijet events in the SR are further suppressed by requiring $\tilde{\omega }_{\text {min}} > 0.3$. Requirements to suppress QCD events are applied in the SR only for ${{\textrm{t}} {}{\overline{{{{\textrm{t}}}}}}} {\textrm{H}}$ categories, and to both SR and CRs in the VH categories in order to ensure good correspondence amongst the regions. The selections applied to $\tilde{\omega }_{\text {min}}$ and $|\Delta \phi _\text {min}({{\vec p}_{\textrm{T}}^{\hspace{1.0pt}\text {miss}}, \vec {p}_\textrm{T,1234})} |$ are not applied in the CRs used for background estimation of the ${{\textrm{t}} {}{\overline{{{{\textrm{t}}}}}}} {\textrm{H}}$ categories, where the hadronic recoil does not stem from jet mismeasurement. This is to increase event counts in the CRs, particularly in the boosted ${{\textrm{t}} {}{\overline{{{{\textrm{t}}}}}}} {\textrm{H}}$ categories.

The hadronic recoil in ${{\textrm{t}} {}{\overline{{{{\textrm{t}}}}}}} {\textrm{H}}$ production is closely aligned with the direction of the Higgs boson typically. In t $\overline{{{{\textrm{t}}}}}$ events, the ${\vec p}_{\textrm{T}}^{\hspace{1.0pt}\text {miss}}$ is usually parallel or antiparallel to the direction of the leading b jet, as the t quarks are produced back-to-back. Therefore, the angles between the direction of the hadronic recoil and the leading or subleading jet or b jet ${\vec p}_{\textrm{T}}$ directions provide additional features for t $\overline{{{{\textrm{t}}}}}$ background suppression in the resolved ${{\textrm{t}} {}{\overline{{{{\textrm{t}}}}}}} {\textrm{H}}$ categories. The angular variables $|\Delta \phi (\overrightarrow{\text {recoil}}, \vec {p}^{~\text {j}}_\textrm{T,1}) |$, $|\Delta \phi (\overrightarrow{\text {recoil}}, \vec {p}^{~\text {b}}_\textrm{T,1}) |$, and $|\Delta \phi (\overrightarrow{\text {recoil}},\vec {p}^{~\text {b}}_\textrm{T,2}) |$ are the most sensitive discriminators between ${{\textrm{t}} {}{\overline{{{{\textrm{t}}}}}}} {\textrm{H}}$ and t $\overline{{{{\textrm{t}}}}}$. The selection based on these angular variables has been optimised by maximising the combined expected sensitivity of the ${{\textrm{t}} {}{\overline{{{{\textrm{t}}}}}}} {\textrm{H}}$ analysis and is summarised in Table 2.

## Control regions and background estimation

The analysis makes use of the ${\upmu } + \text {jets}$ and ${\textrm{e}} + \text {jets}$ CRs to estimate $\ell _{\text {lost}}$ background contributions, which are mainly from ${{\textrm{t}} {}{\overline{{{{\textrm{t}}}}}}} + \text {jets}$, single t quark, and ${\textrm{W}} + \text {jets}$ events. The background contributions from ${\textrm{Z}} \rightarrow \text {inv}$, which include ${\textrm{ZZ}}$, ${{{\textrm{t}} {}{\overline{{{{\textrm{t}}}}}}} {\textrm{Z}}}$, and Drell-Yan (DY) contributions, are estimated from the ${\upmu \upmu }\,+$ jets, ee $+$ jets, and ${\upgamma } + \text {jets}$ CRs. Hadronic backgrounds in the SR such as QCD multijet contributions are estimated using a transfer factor method applied to a QCD enriched sideband CR.

### Estimation of $\ell _{\text {lost}}$ and ${\textrm{Z}} \rightarrow \text {inv}$ backgrounds

The ${\upmu } + \text {jets}$ (${\textrm{e}} + \text {jets}$) CR is defined by requiring exactly one tightly-isolated muon (electron) with $p_{\textrm{T}} >20 (40)\,\text {Ge}\hspace{-.08em}\text {V}$. Both CRs require ${50< m_\textrm{T}^{\ell } < 110\,\text {Ge}\hspace{-.08em}\text {V}}$. The single-lepton CRs are used to constrain the $\ell _{\text {lost}}$ background, which is the main source of background in the ${{\textrm{t}} {}{\overline{{{{\textrm{t}}}}}}} {\textrm{H}}$ and VH  2j2b categories. In the ${{\textrm{t}} {}{\overline{{{{\textrm{t}}}}}}} {\textrm{H}}$ category, the $\ell _{\text {lost}}$ contribution arises mainly from t $\overline{{{{\textrm{t}}}}}$, single t quark, and ${{{\textrm{t}} {}{\overline{{{{\textrm{t}}}}}}} {\textrm{V}}}$ processes, while in the VH category it is from W  $+$ jet events.

In the ${\upmu \upmu }\,+$ jets (ee $+$ jets) CR, one tightly-isolated muon (electron) with ${p_{\textrm{T}} >20~(40)\,\text {Ge}\hspace{-.08em}\text {V}}$, and one loose muon (electron) with the opposite charge and $p_{\textrm{T}} >10 (10)\,\text {Ge}\hspace{-.08em}\text {V}$ are required with invariant mass, $m_{\upmu \upmu }$ ($m_{\textrm{ee}}$), compatible with a Z boson. For the ${{\textrm{t}} {}{\overline{{{{\textrm{t}}}}}}} {\textrm{H}}$ (VH) category, the invariant mass is required to be between 75 and 105 (60 and 120)$\,\text {Ge}\hspace{-.08em}\text {V}$. The processes ${\textrm{Z}} \rightarrow {{\upnu }} {{{{\upnu }}}}$ and ${{\textrm{Z}} \rightarrow \ell \ell }$ are kinematically nearly identical, largely due to lepton universality, hence the dilepton regions can be used to constrain the ${\textrm{Z}} \rightarrow \text {inv}$ background and minimise theoretical uncertainties. This is important for the ${\textrm{Z}} \rightarrow \text {inv}$ background, which dominates the VH category and contributes to the ${{\textrm{t}} {}{\overline{{{{\textrm{t}}}}}}} {\textrm{H}}$ category especially at high hadronic recoil. In the ${{\textrm{t}} {}{\overline{{{{\textrm{t}}}}}}} {\textrm{H}}$ category, events are selected for which ${\Delta \phi (\overrightarrow{\text {recoil}}, \vec {p}_{\textrm{T},\text {track}}^{\text {miss}})>\pi /2}$, which reduces the ${{\textrm{t}} {}{\overline{{{{\textrm{t}}}}}}} + \text {jets}$ background and favours DY production in the dilepton CRs.

The ${\upgamma } + \text {jets}$ CR is used for background estimation in the VH category only, and requires exactly one loose photon with ${p_{\textrm{T}} >230\,\text {Ge}\hspace{-.08em}\text {V}}$. This region is used to constrain the ${\textrm{Z}} \rightarrow \text {inv}$ background as the event kinematics and topologies are similar for ${\textrm{Z}} + \text {jets}$ and ${\upgamma } + \text {jets}$ events, improving the sensitivity to the VH signal primarily at high hadronic recoil compared to the dilepton CRs because of the larger number of events.

Photons can usually be discriminated from other sources of ECAL deposits using the properties of the deposits themselves, such as isolation in ECAL and HCAL, or the shape of the electromagnetic showers. However, occasionally other particles will be incorrectly identified as photons, for example where a jet is misidentified as a photon in QCD multijet events. In order to estimate the contribution from misidentified photons in the ${\upgamma } + \text {jets}$ CR, a purity measurement is performed. The purity is defined as the fraction of reconstructed photon candidates that correspond to genuine isolated photons originating from the PV in the event. The photon purity is measured in data based on the lateral width $\sigma _{\eta \eta }$ [85], which parametrises the shape of the energy deposit associated with the photon in the ECAL. The characteristic $\sigma _{\eta \eta }$ distribution from genuine photons peaks at $\sigma _{\eta \eta } <1$, while the distribution due to misidentified photons possesses a less pronounced peak with a much broader decline for $\sigma _{\eta \eta } >1$. A template fit to the $\sigma _{\eta \eta }$ distribution is performed, where for genuine photons simulated ${\upgamma } + \text {jets}$ events are used to build the signal templates, while for misidentified photons a data sample enriched in misidentified photon events is obtained by inverting the isolation requirements in the ${\upgamma } + \text {jets}$ CR. The purity is defined as the fraction of genuine photons extracted from the fit that pass the $\sigma _{\eta \eta }$ selection. The photon purity is measured separately in bins of $p_{\textrm{T}}^{{\upgamma }}$ and for each data-taking period and varies between 1.5 and 4.5%. The contamination is the fraction of misidentified photons in the ${\upgamma } + \text {jets}$ CR, and is estimated at around 4% for ${p_{\textrm{T}}^{{\upgamma }}>200\,\text {Ge}\hspace{-.08em}\text {V}}$. The QCD multijet contribution in the ${\upgamma } + \text {jets}$ CR is then estimated by weighting events in data for each $p_{\textrm{T}}^{{\upgamma }}$ bin by the corresponding contamination. A 25% systematic uncertainty is attributed to the QCD multijet background normalisation, and is estimated by performing the procedure for different $\sigma _{\eta \eta }$ binning in the template fit, which accounts for any mismodelling of the simulated $\sigma _{\eta \eta }$ distribution. The statistical uncertainty in the photon purity estimate in each $p_{\textrm{T}}^{{\upgamma }}$ bin is found to be much smaller than the systematic one. The full requirements for the analysis CRs are shown in Table 3.

**Table 3:** Summary of all CR requirements, excluding selections suppressing the QCD multijet background, and excluding the requirement of ${\Delta \phi (\overrightarrow{\text {recoil}},\vec {p}_{\textrm{T},\text {track}}^{\text {miss}})>\pi /2}$ applied to the ${{\textrm{t}} {}{\overline{{{{\textrm{t}}}}}}} {\textrm{H}}$ category in the dilepton CRs. No mass requirements are imposed in the ${\upgamma } + \text {jets}$

Control region	Category	$n_{\text {object}}$ reqs.	Mass reqs. ($\text {Ge}\hspace{-.08em}\text {V}$)	$p_{\textrm{T}}$ reqs. ($\text {Ge}\hspace{-.08em}\text {V}$)
${\upmu } + \text {jets}$	${{\textrm{t}} {}{\overline{{{{\textrm{t}}}}}}} {\textrm{H}}$	$n_{\upmu } = 1$	$50< m_{\textrm{T}} ^{\upmu } < 110$	$p^{~{\upmu }}_{\textrm{T,1}} > 20$
	VH			
${\textrm{e}} + \text {jets}$	${{\textrm{t}} {}{\overline{{{{\textrm{t}}}}}}} {\textrm{H}}$	$n_{{\textrm{e}}} = 1$	$50< m_{\textrm{T}} ^{{\textrm{e}}} < 110$	$p^{~{\textrm{e}}}_{\textrm{T,1}} > 40$
	VH			
${\upmu \upmu }\,\,+\,$ jets	${{\textrm{t}} {}{\overline{{{{\textrm{t}}}}}}} {\textrm{H}}$	$n_{{\upmu }} = 2$	$75< m_{\upmu \upmu } < 105$	$p^{~{\upmu }}_{\textrm{T,1}} > 20$, $p^{~{\upmu }}_{\textrm{T,2}} > 10$
	VH		$60< m_{\upmu \upmu } < 120$	
ee $+$ jets	${{\textrm{t}} {}{\overline{{{{\textrm{t}}}}}}} {\textrm{H}}$	$n_{{\textrm{e}}} = 2$	$75< m_{\textrm{ee}} < 105$	$p^{\mathrm {~{\textrm{e}}}}_{\textrm{T,1}} > 40$, $p^{\mathrm {~{\textrm{e}}}}_{\textrm{T,2}} > 10$
	VH		$60< m_{\textrm{ee}} < 120$	
${\upgamma } + \text {jets}$	VH	$n_{{\upgamma }} = 1$	–	$p_{\textrm{T}}^{{\upgamma }} > 230$

### Residual backgrounds from QCD multijet production

The event selection aims to reduce background contributions from QCD multijet production as much as possible by requiring ${|\Delta \phi _\text {min}({{\vec p}_{\textrm{T}}^{\hspace{1.0pt}\text {miss}}, \vec {p}_\textrm{T,1234})} | >0.5}$ and ${\tilde{\omega }_{\text {min}} >0.3}$, although a QCD multijet background enriched sideband is used to estimate any remaining background contribution with the help of a transfer factor between sideband and SR, which is derived from simulation. The sideband is defined with an identical selection to that of the SR, but with an inversion on the requirements on $|\Delta \phi _\text {min}({{\vec p}_{\textrm{T}}^{\hspace{1.0pt}\text {miss}}, \vec {p}_\textrm{T,1234})} |$ and $\tilde{\omega }_{\text {min}}$, such that $|\Delta \phi _\text {min}({{\vec p}_{\textrm{T}}^{\hspace{1.0pt}\text {miss}}, \vec {p}_\textrm{T,1234})} | <0.5$ and more stringently $\tilde{\omega }_{\text {min}} < 0.2$. The criteria for $\tilde{\omega }_{\text {min}}$ is determined by optimising the sideband to be as QCD multijet-enriched as possible while ensuring the SR has negligible QCD multijet background. For the VH category, the $m_{\text {jj}}$ requirement is also inverted in order to have the sideband sufficiently populated.

The SRs in both the ${{\textrm{t}} {}{\overline{{{{\textrm{t}}}}}}} {\textrm{H}}$ and VH categories suffer from limited simulated QCD multijet event counts, so it is not possible to reliably define a transfer factor for each SR bin in individual subcategories. Within the statistical precision of the simulated QCD multijet samples, the shape of the hadronic recoil and relative population of the ${{\textrm{t}} {}{\overline{{{{\textrm{t}}}}}}} {\textrm{H}}$ subcategories are observed not to depend on $\tilde{\omega }_{\text {min}}$ and $|\Delta \phi _\text {min}({{\vec p}_{\textrm{T}}^{\hspace{1.0pt}\text {miss}}, \vec {p}_\textrm{T,1234})} |$. Therefore, the expected QCD sideband yields are integrated over all ${{\textrm{t}} {}{\overline{{{{\textrm{t}}}}}}} {\textrm{H}}$ subcategories and hadronic recoil intervals, and over hadronic recoil intervals for each VH category, in the sideband and SR, to construct the transfer factors. The resulting hadronic recoil distributions are used to predict the relative QCD multijet background in each subcategory and hadronic recoil interval.

The estimated QCD multijet background yield in the ${{\textrm{t}} {}{\overline{{{{\textrm{t}}}}}}} {\textrm{H}}$ SR for subcategory *i* and hadronic recoil interval *j*, $N^{\text {QCD, SR}_{{{\textrm{t}} {}{\overline{{{{\textrm{t}}}}}}} {\textrm{H}} }}_{i,j}$, is given by

2 $$\begin{aligned} N^{\text {QCD, SR}_{{{\textrm{t}} {}{\overline{{{{\textrm{t}}}}}}} {\textrm{H}} }}_{i,j}= & {} \sum _p \sum _q \Bigg (N^{\text {data, CR}_{{{\textrm{t}} {}{\overline{{{{\textrm{t}}}}}}} {\textrm{H}} }}_{p,q}- N^{\text {EW, CR}_{{{\textrm{t}} {}{\overline{{{{\textrm{t}}}}}}} {\textrm{H}} }}_{p,q}\Bigg ) \text {TF}^{{{\textrm{t}} {}{\overline{{{{\textrm{t}}}}}}} {\textrm{H}} }_{\text {QCD}} f^{{{\textrm{t}} {}{\overline{{{{\textrm{t}}}}}}} {\textrm{H}} }_{c_{i}} f^{{{\textrm{t}} {}{\overline{{{{\textrm{t}}}}}}} {\textrm{H}} }_{m_{j}}, \quad \end{aligned}$$

where EW refers to processes that are not QCD multijet, summation indices *p* and *q* are the subcategory and hadronic recoil bins, respectively, $\text {TF}^{{{\textrm{t}} {}{\overline{{{{\textrm{t}}}}}}} {\textrm{H}} }_{\text {QCD}}$ is the QCD multijet simulation transfer factor defined as the ratio between the expected QCD multijet background contribution in the SR and the sideband, and $f^{{{\textrm{t}} {}{\overline{{{{\textrm{t}}}}}}} {\textrm{H}} }_{c_{i}}$ and $f^{{{\textrm{t}} {}{\overline{{{{\textrm{t}}}}}}} {\textrm{H}} }_{m_{j}}$ are the fractions of simulated QCD multijet events in each subcategory and hadronic recoil bin, respectively.

In the VH category, the sideband regions are defined for each subcategory, as the number of simulated QCD multijet events is sufficient to derive the hadronic recoil fractions $f_{m_{j}}$ separately for each subcategory. The method is otherwise analogous to that of ${{\textrm{t}} {}{\overline{{{{\textrm{t}}}}}}} {\textrm{H}}$, given by Eq. 2.

The results of the QCD prediction aggregated over data sets from the 2016–2018 period are found to be small in comparison to background contributions from $\ell _{\text {lost}}$ and ${\textrm{Z}} \rightarrow \text {inv}$ processes. In addition to the statistical uncertainties, a 100% systematic uncertainty is assigned to the predicted background yields from QCD multijet production. The actual uncertainty in the QCD prediction is measured at around 50%, derived by calculating the QCD contribution in the entire ${{\textrm{t}} {}{\overline{{{{\textrm{t}}}}}}} {\textrm{H}}$ category for a signal-depleted validation region analogous to the SR but requiring $0.2<\tilde{\omega }_{\text {min}} <0.3$ and ${|\Delta \phi _\text {min}({{\vec p}_{\textrm{T}}^{\hspace{1.0pt}\text {miss}}, \vec {p}_\textrm{T,1234})} |}>0.5$, and comparing the estimate to data. It is inflated to 100% to be more conservative when handling the individual ${{\textrm{t}} {}{\overline{{{{\textrm{t}}}}}}} {\textrm{H}}$ subcategories that are limited by event counts at larger hadronic recoil, which was found to have negligible impact on the final fit.

## Statistical interpretation

A maximum likelihood fit method is used to obtain an upper limit on ${\mathcal {B}({\textrm{H}} \rightarrow \text {inv})}$. The fit is performed simultaneously across each year, region, category, and hadronic recoil interval, with systematic uncertainties acting as nuisance parameters in the fit correlated to varying degrees across year and category.

### Likelihood model

The limits on ${\mathcal {B}({\textrm{H}} \rightarrow \text {inv})}$ are extracted via a simultaneous binned maximum likelihood fit to the hadronic recoil distributions obtained in the SR and CRs. The likelihood can be written as

3 $$\begin{aligned} \mathcal {L}=\mathcal {L}_{\text {SR}}~\mathcal {L}_{{\upmu }}~\mathcal {L}_{{\textrm{e}}} ~\mathcal {L}_{{\upmu } {\upmu }}~\mathcal {L}_{\textrm{ee}}~\mathcal {L}_{{\upgamma }}, \end{aligned}$$

where $\mathcal {L}_{\text {SR}}$ is the likelihood function for the SR (boosted ${{\textrm{t}} {}{\overline{{{{\textrm{t}}}}}}} {\textrm{H}}$, resolved ${{\textrm{t}} {}{\overline{{{{\textrm{t}}}}}}} {\textrm{H}}$, VH), and $\mathcal {L}_{{\upmu }}$, $\mathcal {L}_{{\textrm{e}}}$, $\mathcal {L}_{{\upmu } {\upmu }}$, $\mathcal {L}_{{\textrm{ee}}}$, and $\mathcal {L}_{{\upgamma }}$ designate the likelihood functions for the ${\upmu } + \text {jets}$, ${\textrm{e}} + \text {jets}$, ${\upmu \upmu }+$ jets, ee $+$ jets, and ${\upgamma } + \text {jets}$ CRs, respectively. The likelihood function for the SR is defined as

4 $$\begin{aligned} \mathcal {L}_\text {SR}=\prod _{\text {cat}=i}^{n_{\text {cat}}} \prod _{\text {recoil}=j(i)}^{n_{\xi (i)}}\text {Poisson}\left( n_\text {obs}^{i,j}\mid n^{i,j}_\text {pred}\right) , \end{aligned}$$

with

5 $$\begin{aligned} \begin{aligned} n^{i,j}_\text {pred}&= \hat{\mu } s^{i,j} \rho _{s}^{i,j}\\&\quad + b^{i,j}_{\ell _{\text {lost}}} I^{i,j} \rho _{\ell _{\text {lost}}}^{i,j}\\&\quad + b^{i,j}_{{\textrm{Z}} \rightarrow \text {inv}} L^{i,j} \rho _{{\textrm{Z}} \rightarrow \text {inv}}^{i,j}\\&\quad + b^{i,j}_\text {QCD} \rho ^{i,j}_\text {QCD}, \end{aligned} \end{aligned}$$

where the symbols are defined in Table 4. The signal strength, $\hat{\mu }$, is interpreted as the maximum likelihood estimator for ${\mathcal {B}({\textrm{H}} \rightarrow \text {inv})}$, where the signal prediction assumes that ${\mathcal {B}({\textrm{H}} \rightarrow \text {inv})} =1$. The fit also includes additional free parameters $I^{i,j}$ and $L^{i,j}$, which depend on category *i*, hadronic recoil bin *j*, and the number of recoil bins in each category $n_{\xi (i)}$. The first of these parameters, $I^{i,j}$, simultaneously scales the normalisation of the $\ell _{\text {lost}}$ background in the SR and the sum of the ${\textrm{W}} + \text {jets}$, t $\overline{{{{\textrm{t}}}}}$  $+$ jets, and single t quark backgrounds, $\text {X}^{i,j}_{{\textrm{t}},{\textrm{W}}}$, in the ${\upmu } + \text {jets}$ and ${\textrm{e}} + \text {jets}$ CRs. The second of these parameters, $L^{i,j}$, simultaneously scales the normalisation of the ${\textrm{Z}} \rightarrow \text {inv}$ background in the SR (Z(${{{\upnu }} {{{{\upnu }}}}}$) $+$ jets and ${{{\textrm{t}} {}{\overline{{{{\textrm{t}}}}}}} {\textrm{Z}} ({{\upnu }} {{{{\upnu }}}})}$) and the sum of the $\upgamma$  $+$ jets, DY $+$ jets, ${{{\textrm{t}} {}{\overline{{{{\textrm{t}}}}}}} {\textrm{Z}}}$ $+$ jets, and multiboson backgrounds, $\text {X}^{i,j}_{{\textrm{Z}}/{\upgamma }}$, in the ${\upmu \upmu } +$ jets, ee $+$ jets, and ${\upgamma } + \text {jets}$ CRs.

**Table 4:** Meaning of the symbols used in Eqs. 4 and 5 that define the likelihood function

Symbol	Meaning
$\hat{\mu }$	Signal strength estimator of ${\mathcal {B}({\textrm{H}} \rightarrow \text {inv})}$
$s^{i,j}$	Simulation predicted number of signal events in bin *i*, *j* of the SR
$\rho _{s}^{i,j}$	Systematic uncertainties affecting signal prediction in bin *i*, *j* of the SR
$b^{i,j}_{\ell _{\text {lost}}}$	Simulation predicted number of $\ell _{\text {lost}}$ events in bin *i*, *j* of the SR
$I^{i,j}$	Normalisation parameter for the $\ell _{\text {lost}}$ estimation in bin *i*, *j*
$\rho _{\ell _{\text {lost}}}^{i,j}$	Systematic uncertainties affecting the $\ell _{\text {lost}}$ background in bin *i*, *j* of the SR
$b^{i,j}_{{\textrm{Z}} \rightarrow \text {inv}}$	Simulation predicted number of ${\textrm{Z}} \rightarrow \text {inv}$ events in bin *i*, *j* of the SR
$L^{i,j}$	Normalisation parameter for the ${\textrm{Z}} \rightarrow \text {inv}$ estimation in bin *i*, *j*
$\rho _{{\textrm{Z}} \rightarrow \text {inv}}^{i,j}$	Systematic uncertainties affecting the ${\textrm{Z}} \rightarrow \text {inv}$ background in bin *i*, *j* of the SR
$b^{i,j}_\text {QCD}$	Predicted number of QCD events in bin *i*, *j* of the SR
$\rho ^{i,j}_\text {QCD}$	Systematic uncertainties of the QCD component in bin *i*, *j* of the SR

The likelihood for the ${\upmu } + \text {jets}$ and ${\textrm{e}} + \text {jets}$ CRs is given by6and for the ${\upmu \upmu }\,+$ jets, ee $+$ jets, and ${\upgamma } + \text {jets}$ CRs is given by

7 $$\begin{aligned}{} & {} \mathcal {L}_{\upmu \upmu ,{\textrm{ee}},{\upgamma }}= \prod _{\text {cat}=i}^{n_\text {cat}}\prod _{\text {recoil}=j(i)}^{n_{\xi (i)}} \nonumber \\{} & {} \quad \times \text {Poisson}\left( n_\text {obs}^{i,j}\mid \text {X}^{i,j}_{{\textrm{Z}}/{\upgamma }}L^{i,j}\rho _{{\textrm{Z}}/{\upgamma }}^{i,j} + \text {X}^{i,j}_\text {other} \rho _\text {other}^{i,j}\right) , \end{aligned}$$

where X$^{i,j}$ is the sum of background yields, and $\rho ^{i,j}$ refers to the associated systematic uncertainty.

Because of the low event counts in the dilepton CRs, the subcategory yields are summed into the boosted and resolved ${{\textrm{t}} {}{\overline{{{{\textrm{t}}}}}}} {\textrm{H}}$ categories. For the boosted ${{\textrm{t}} {}{\overline{{{{\textrm{t}}}}}}} {\textrm{H}}$ category, the ${\upmu \upmu }+$ jets and ee $+$ jets CR yields are summed together to form a single $\ell \ell + \text {jets}$ CR. Furthermore, in the boosted and resolved ${{\textrm{t}} {}{\overline{{{{\textrm{t}}}}}}} {\textrm{H}}$ subcategories, $I^{i,j}$ are shared across subcategories, therefore *i* takes only two values corresponding to the boosted and resolved ${{\textrm{t}} {}{\overline{{{{\textrm{t}}}}}}} {\textrm{H}}$ classes.

### Systematic uncertainties

The model on which the maximum likelihood fit is based is inclusive of experimental and theoretical uncertainties. These are modelled as nuisance parameters, which are typically constrained by a template fit where there is a dependence on the hadronic recoil distribution, but are otherwise constrained by a log-normal distribution for those that affect the overall normalisation of a given process.

Theoretical uncertainties related to the PDF parameters and missing higher order corrections in the QCD and EW perturbative expansions are estimated by following the procedure outlined in Ref. [60] for ${{\textrm{t}} {}{\overline{{{{\textrm{t}}}}}}} {\textrm{H}}$ and VH processes, and in Ref. [86] for ${\textrm{V}} + \text {jets}$ and ${\upgamma } + \text {jets}$ processes. Systematic uncertainties related to the PDF, and the renormalisation and factorisation scales, are treated as independent nuisance parameters but are correlated across years in the fit.

A photon normalisation uncertainty of 40% is included in the ${\upgamma } + \text {jets}$ CR, to cover uncertainties in the translation between the ${\upgamma } + \text {jets}$ and ${{\textrm{Z}} \rightarrow \ell \ell }$ yields, and is only correlated between 2017 and 2018 samples given the ${\upgamma } + \text {jets}$ sample for 2016 is generated with a different tune.

Data-derived correction factors are applied to simulated events containing b, t, and W jets, and therefore the systematic uncertainties due to the limited precision in these corrections are propagated to the simulated samples. These are referred to as tagging uncertainties, and also account for the uncertainties in the tagging efficiencies and misidentification probabilities. The tagging methods and uncertainty propagation are consistent between years, and therefore are correlated across years in the fit.

The uncertainty in the combined PF $p_{\textrm{T}} ^\text {miss}$ and $H_\textrm{T}^\text {miss}$ trigger efficiency is computed using the ${\upmu } + \text {jets}$ and ${\upmu \upmu } +$ jets CRs. These are independent of the $p_{\textrm{T}} ^\text {miss}$ and $H_\textrm{T}^\text {miss}$ data sets in the SR, ensuring an unbiased measurement of the uncertainty. This uncertainty is measured at 2%, and is applied independently in each year of data-taking due to variations in the trigger performance. The same uncertainty is measured in the electron and photon trigger efficiency, and is similarly uncorrelated between years. An additional trigger inefficiency uncertainty due to the mistiming of ECAL trigger inputs detailed in Sect. 5.1 is applied to the data-taking years 2016 and 2017.

The uncertainty in the integrated luminosity varies between 1.2–2.5% depending on the data-taking year, with an overall uncertainty of 1.6% for the 2016–2018 period [46–48]. The uncertainty is applied with correlated and uncorrelated components across years.

The uncertainties considered in the analysis are presented in Table 5 with the pre-fit ranges corresponding to the maximum and minimum deviations of the event yields from their nominal values across each region, year of data-taking, category, recoil bin, and all SM background processes, when the respective systematic uncertainty is changed within ±1 standard deviation. Systematic uncertainties not specified above are typically assumed to be uncorrelated from year to year when performing the fit. Those for which the source of the systematic uncertainty is identical for each year are treated as correlated. All systematic uncertainties are correlated across regions.

The overall experimental uncertainty is found to be dominated by W tagging for the ${{\textrm{t}} {}{\overline{{{{\textrm{t}}}}}}} {\textrm{H}}$ and b tagging for the VH categories in the SR. The lepton and photon candidate efficiencies for identification, isolation, and reconstruction, and uncertainties in the JER, JES, and trigger efficiencies also make significant contributions. The theoretical uncertainty is dominated by variations in the renormalisation scale, factorisation scale, and PDF for ${\textrm{V}} + \text {jets}$ processes, although these are particularly sensitive to the high exclusive jet multiplicity characterising the ${{\textrm{t}} {}{\overline{{{{\textrm{t}}}}}}} {\textrm{H}}$ and VH categories.

**Table 5:** The ranges corresponding to the maximum and minimum deviations of the event yields from their nominal values, provided where applicable across each region, year of data-taking, category, recoil bin, and all SM background processes, when the respective systematic uncertainty is changed within ±1 standard deviation

Systematic uncertainties on background yields (pre-fit)	Signal region		$\ell + \text {jets}$		$\ell \ell + \text {jets}$		${\upgamma } + \text {jets}$
	${{\textrm{t}} {}{\overline{{{{\textrm{t}}}}}}} {\textrm{H}}$ cat.	VH cat.	${{\textrm{t}} {}{\overline{{{{\textrm{t}}}}}}} {\textrm{H}}$ cat.	VH cat.	${{\textrm{t}} {}{\overline{{{{\textrm{t}}}}}}} {\textrm{H}}$ cat.	VH cat.	VH cat.
*Theoretical uncertainties*							
Fact. scale ${\textrm{V}} + \text {jets}$ (QCD)	<1.0-7.7 %	<1.0-19 %	<1.0-2.6 %	<1.0-11 %	<1.0-20 %	<1.0-22 %	6.0 %
Ren. scale ${\textrm{V}} + \text {jets}$ (QCD)	<1.0-7.2 %	<1.0-8.6 %	<1.0-3.6 %	<1.0-10 %	<1.0-14 %	2.0-11 %	12 %
PDF ${\textrm{V}} + \text {jets}$	<1.0-9.1 %	2.0-23 %	<1.0-3.1 %	<1.0-15 %	<1.0-23 %	<1.0-26 %	8.0 %
Ren. & Fact. scale ${{\textrm{t}} {}{\overline{{{{\textrm{t}}}}}}} {\textrm{H}}$ (QCD)	<1.0-1.7 %	<1.0 %	<1.0-1.4 %	<1.0-1.4 %	<1.0 %	<1.0 %	–
Ren. & Fact. scale t $\overline{{{{\textrm{t}}}}}$ (QCD)	7.8-15 %	2.5-9.3 %	6.4-17 %	<1.0-6.3 %	<1.0-5.8 %	<1.0-5.8 %	–
NNLO QCD & NLO EW t quark $p_{\textrm{T}}$ reweighting (inc. PDF)	<1.0-3.1 %	<1.0-1.2 %	<1.0-4.0 %	<1.0-3.9 %	<1.0 %	<1.0 %	–
Ren. & Fact. scale VV (QCD)	<1.0 %	<1.0 %	<1.0 %	<1.0 %	<1.0 %	<1.0 %	<1.0 %
${{\textrm{t}} {}{\overline{{{{\textrm{t}}}}}}} {\textrm{H}}$ & VH cat. cross section (QCD)	5.8-9.2 %	<1.0-3.8 %	–	–	–	–	–
${{\textrm{t}} {}{\overline{{{{\textrm{t}}}}}}} {\textrm{H}}$ & VH cat. cross section (PDF & $\alpha _{s}$)	3.6 %	1.6-1.8 %	–	–	–	–	–
Initial-state radiation	2.0 %	3.0-6.0 %	2.0 %	<1.0-4.2 %	2.0 %	6.0 %	<1.0-4.0 %
Final-state radiation	5.0 %	3.0-5.0 %	2.0-2.2 %	<1.0-3.1 %	4.6-5.0 %	5.0 %	2.0-3.0 %
Photon normalisation	–	–	–	–	–	–	40 %
*Experimental uncertainties*							
Integrated luminosity	1.2-2.5 %	1.2-2.5 %	1.2-2.5 %	1.2-2.5 %	1.2-2.5 %	1.2-2.5 %	1.2-2.5 %
t-tagging	3.2-6.5 %	–	2.1-5.7 %	–	–	–	–
W-tagging	7.8-18 %	–	7.1-18 %	–	–	–	–
b-tagging	8.2-12 %	8.2-22 %	6.5-11 %	2.4-11 %	5.6-8.7 %	1.6-9.6 %	6.6-9.0 %
Electron identification & isolation	–	–	3.7-11 %	4.7-9.6 %	<1.0-15 %	<1.0-20 %	–
Electron reconstruction	–	–	<1.0-1.8 %	<1.0 %	1.0-1.5 %	<1.0-1.4 %	–
Muon identification	–	–	<1.0-1.0 %	<1.0-1.0 %	<1.0-1.8 %	<1.0-1.9 %	–
Muon isolation	–	–	<1.0 %	<1.0 %	<1.0 %	<1.0 %	–
Lepton veto	<1.0 %	<1.0 %	–	–	–	–	–
Photon identification & isolation	–	–	–	–	–	–	2.4-12 %
Photon reconstruction	–	–	–	–	–	–	<1.0 %
Pileup	1.4-8.8 %	<1.0-4.5 %	<1.0-4.8 %	<1.0-4.7 %	<1.0-2.1 %	<1.0-7.9 %	<1.0-3.3 %
Trigger inefficiency	<1.0-12 %	<1.0-1.4 %	<1.0-3.4 %	<1.0-2.4 %	<1.0-1.6 %	<1.0-1.5 %	<1.0-0.3 %
Trigger	2.0 %	2.0 %	2.0 %	2.0 %	2.0 %	2.0 %	2.0 %
Tau lepton veto	<1.0 %	<1.0 %	<1.0-1.0 %	<1.0-2.4 %	<1.0 %	<1.0 %	<1.0 %
JER	2.4-3.6 %	<1.0-1.1 %	1.7-3.0 %	<1.0-1.5 %	<1.0-3.5 %	<1.0-1.4 %	<1.0-2.9 %
JES	<1.0-6.3 %	<1.0-2.9 %	<1.0-5.0 %	<1.0-2.2 %	<1.0-6.7 %	<1.0-2.8 %	<1.0-3.8 %
QCD prediction	100 %	100 %	–	–	–	–	–

## Results

**Fig. 2:**
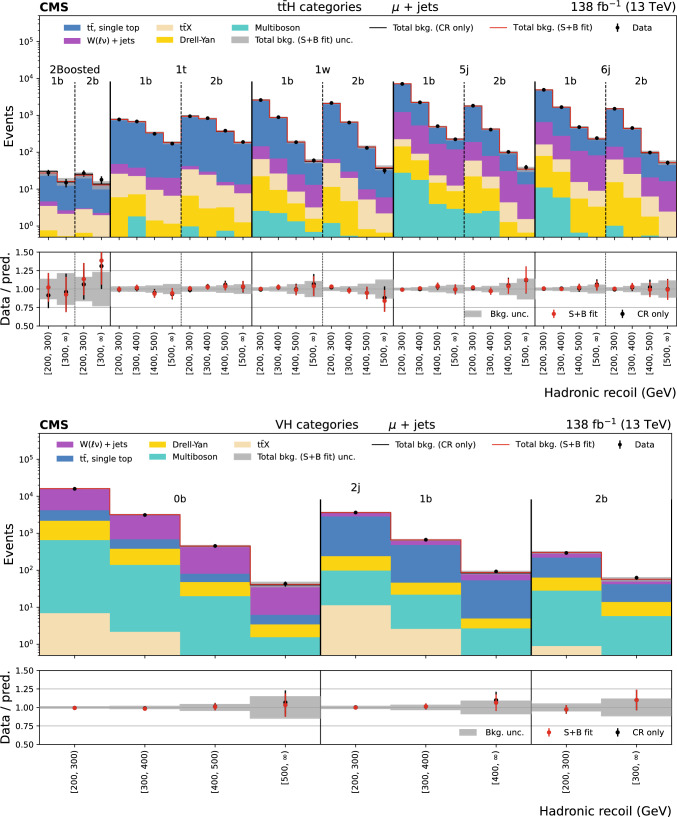
Distributions of hadronic recoil in the ${{\textrm{t}} {}{\overline{{{{\textrm{t}}}}}}} {\textrm{H}}$ (upper plot) and VH (lower plot) categories for the ${\upmu } + \text {jets}$ CR. The black histogram shows the total background (bkg.) prediction from a CR only, B-only fit, while the red histogram shows the yields from a CR + SR S + B fit. The uncertainty in the predicted background (Bkg. unc.) accounts for both systematic and simulated statistical contributions

**Fig. 3:**
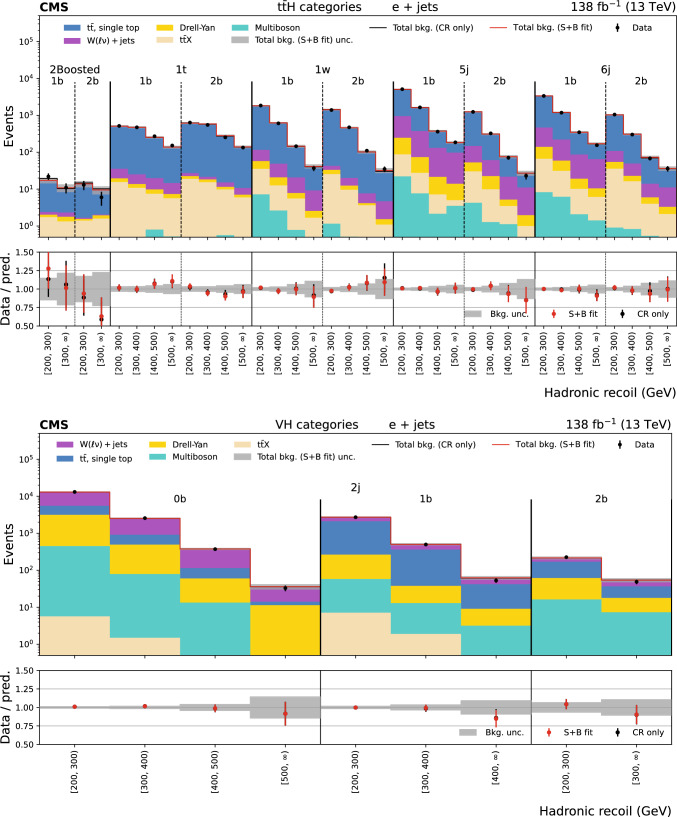
Distributions of hadronic recoil in the ${{\textrm{t}} {}{\overline{{{{\textrm{t}}}}}}} {\textrm{H}}$ (upper plot) and VH (lower plot) categories for the ${\textrm{e}} + \text {jets}$ CR. The black histogram shows the total background (bkg.) prediction from a CR only, B-only fit, while the red histogram shows the yields from a CR + SR S + B fit. The uncertainty in the predicted background (Bkg. unc.) accounts for both systematic and simulated statistical contributions

**Fig. 4:**
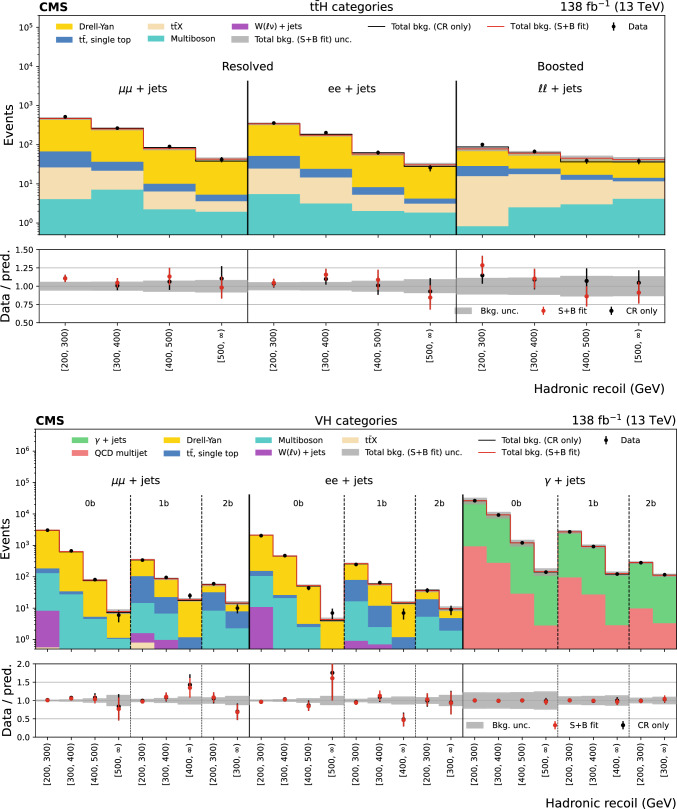
Distributions of hadronic recoil in the ${{\textrm{t}} {}{\overline{{{{\textrm{t}}}}}}} {\textrm{H}}$ category for the ${\upmu \upmu } +$ jets, ee $+$ jets, and $\ell \ell + \text {jets}$ CRs (upper plot), and the VH category for the ${\upmu \upmu }+$ jets, ee $+$ jets, and ${\upgamma } + \text {jets}$ CRs (lower plot). The black histogram shows the total background (bkg.) prediction from a CR only, B-only fit, while the red histogram shows the yields from a CR + SR S + B fit. The uncertainty in the predicted background (Bkg. unc.) accounts for both systematic and simulated statistical contributions

**Fig. 5:**
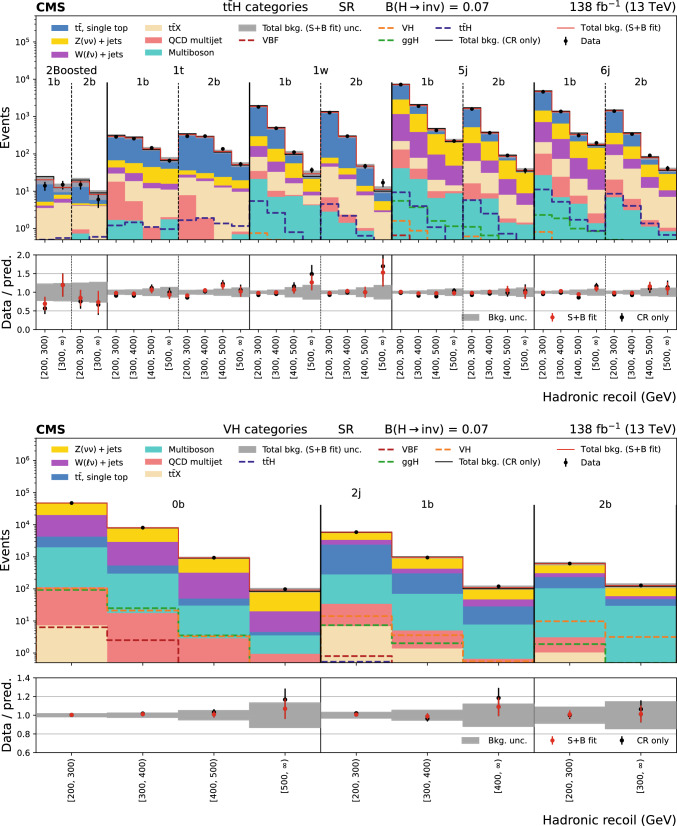
Distributions of hadronic recoil in the ${{\textrm{t}} {}{\overline{{{{\textrm{t}}}}}}} {\textrm{H}}$ (upper plot) and VH (lower plot) categories for the SR, showing the signal contributions from ${{\textrm{t}} {}{\overline{{{{\textrm{t}}}}}}} {\textrm{H}}$, VH, ggH, and VBF weighted by ${\mathcal {B}({\textrm{H}} \rightarrow \text {inv})} =0.07$. The black histogram shows the total background (bkg.) prediction from a CR only, B-only fit, while the red histogram shows the yields from a CR + SR S + B fit. The uncertainty in the predicted background (Bkg. unc.) accounts for both systematic and simulated statistical contributions

The hadronic recoil distributions across all ${{\textrm{t}} {}{\overline{{{{\textrm{t}}}}}}} {\textrm{H}}$ and VH subcategories are shown in Figs. 2, 3, 4 and 5. The predicted background yield from the fit to the CRs only is shown with the result of a fit including the data in the SR. The agreement between the data and simulation is presented below each distribution, with the uncertainty in the predicted background uncertainty (Bkg. unc.) accounting for both systematic and simulated statistical contributions. Figures 2 (3) shows the ${\upmu } + \text {jets}$ (${\textrm{e}} + \text {jets}$) CR yields for the ${{\textrm{t}} {}{\overline{{{{\textrm{t}}}}}}} {\textrm{H}}$ and VH categories, respectively, aggregated over 2016–2018. In these CRs, $\ell _{\text {lost}}$ background from t $\overline{{{{\textrm{t}}}}}$, ${\textrm{W}} \rightarrow \ell {{\upnu }}$, and single t quark production dominates, with smaller contributions from multiboson and ${{{\textrm{t}} {}{\overline{{{{\textrm{t}}}}}}} {\textrm{X}}}$ processes. The ${\upmu \upmu }+$ jets, ee $+$ jets, $\ell \ell + \text {jets}$ (only for ${{\textrm{t}} {}{\overline{{{{\textrm{t}}}}}}} {\textrm{H}}$), and ${\upgamma } + \text {jets}$ (only for VH) CR distributions used for the prediction of backgrounds stemming from ${\textrm{Z}} \rightarrow \text {inv}$ decays are shown in Fig. 4 for 2016–2018. In addition, the total SM background prediction in the SR, consisting of $\ell _{\text {lost}}$, ${\textrm{Z}} \rightarrow \text {inv}$, and QCD backgrounds, is shown for the ${{\textrm{t}} {}{\overline{{{{\textrm{t}}}}}}} {\textrm{H}}$ and VH category in Fig. 5. The SR distributions contain all the Higgs boson production modes in the fitted ${\mathcal {B}({\textrm{H}} \rightarrow \text {inv})}$ signal, including the ggH and VBF contamination in the ${{\textrm{t}} {}{\overline{{{{\textrm{t}}}}}}} {\textrm{H}}$ and VH categories, with the prevalence of the ggH process due to its high production cross section. The post-fit event yields for each subcategory and recoil bin in the SR are tabulated in Table 6. For these results, a fit assuming ${\mathcal {B}({\textrm{H}} \rightarrow \text {inv})}$ $=0$ such that only SM background contributions are considered (B-only) is performed simultaneously using only the CRs (CR only), which are independent of the SR, or across both SR and CRs (CR$+$SR). A fit across all regions, including signal and background contributions (S$+$B fit), is also performed, in which the signal contribution is weighted by the best-fit signal strength, ${\mathcal {B}({\textrm{H}} \rightarrow \text {inv})}$. In all cases, uncertainties are inclusive of statistical and systematic contributions.

**Table 6:** Total post-fit yields in the SRs in each recoil bin and analysis category obtained by summing the contributions from the individual data-taking periods. B-only fits are performed for either CR$+$SR or CR only cases. The extracted signal yields from an S$+$B fit are also reported, where the signal strength is weighted by ${{\mathcal {B}({\textrm{H}} \rightarrow \text {inv})} =0.07}$

Subcategory	Hadronic recoil	$\ell _{\text {lost}}$	${\textrm{Z}} \rightarrow \text {inv}$	QCD	Total background		Data	Signal
		CR only	CR only	CR only	CR only	CR + SR		${\mathcal {B}({\textrm{H}} \rightarrow \text {inv})} =0.07$
		B-only fit	B-only fit	B-only fit	B-only fit	B-only fit		S$+$B fit
${{\textrm{t}} {}{\overline{{{{\textrm{t}}}}}}} {\textrm{H}}$ 1t1b	[200, 300)	251.1 ± 9.5	35.2 ± 4.1	23.1 ± 16.8	309.4 ± 19.8	295.5 ± 11.6	288.0 ± 17.0	1.0 ± 0.8
	[300, 400)	235.2 ± 9.5	35.7 ± 5.0	5.2 ± 4.2	276.1 ± 11.5	268.1 ± 9.1	257.0 ± 16.0	1.3 ± 1.0
	[400, 500)	97.5 ± 5.3	27.6 ± 4.9	0.9 ± 0.6	126.1 ± 7.2	135.5 ± 6.7	145.0 ± 12.0	1.0 ± 0.8
	[500, $\infty$)	37.5 ± 2.9	26.1 ± 4.9	0.3 ± 0.3	63.9 ± 5.7	70.1 ± 5.1	66.0 ± 8.1	0.9 ± 0.7
${{\textrm{t}} {}{\overline{{{{\textrm{t}}}}}}} {\textrm{H}}$ 1t2b	[200, 300)	312.5 ± 12.0	19.0 ± 2.2	10.9 ± 8.6	342.4 ± 14.9	328.1 ± 10.5	298.0 ± 17.3	1.4 ± 1.2
	[300, 400)	265.9 ± 10.7	20.2 ± 2.7	2.5 ± 1.7	288.6 ± 11.2	287.1 ± 9.3	299.0 ± 17.3	1.6 ± 1.3
	[400, 500)	93.6 ± 5.1	15.4 ± 2.6	0.4 ± 0.3	109.5 ± 5.7	116.5 ± 5.2	136.0 ± 11.7	1.2 ± 0.9
	[500, $\infty$)	35.4 ± 2.9	13.8 ± 2.5	0.2 ± <0.1	49.4 ± 3.9	52.8 ± 3.5	53.0 ± 7.3	1.0 ± 0.8
${{\textrm{t}} {}{\overline{{{{\textrm{t}}}}}}} {\textrm{H}}$ 1W1b	[200, 300)	1704.6 ± 49.9	190.7 ± 21.2	18.8 ± 16.8	1914.1 ± 56.8	1855.7 ± 41.2	1819.0 ± 42.6	5.7 ± 4.0
	[300, 400)	395.6 ± 15.1	90.2 ± 12.7	4.3 ± 2.9	490.0 ± 19.9	485.0 ± 16.2	486.0 ± 22.0	2.9 ± 1.9
	[400, 500)	56.2 ± 3.9	35.8 ± 6.5	0.8 ± 0.5	92.7 ± 7.7	103.7 ± 7.1	111.0 ± 10.5	0.9 ± 0.6
	[500, $\infty$)	9.9 ± 1.3	13.9 ± 2.9	0.3 ± <0.1	24.1 ± 3.2	29.5 ± 3.0	37.0 ± 6.1	0.4 ± 0.3
${{\textrm{t}} {}{\overline{{{{\textrm{t}}}}}}} {\textrm{H}}$ 1W2b	[200, 300)	1295.8 ± 40.7	53.1 ± 5.7	5.6 ± 3.8	1354.5 ± 41.3	1311.6 ± 29.4	1276.0 ± 35.7	3.9 ± 3.2
	[300, 400)	266.2 ± 11.8	27.2 ± 3.8	1.3 ± 0.9	294.7 ± 12.4	291.3 ± 9.9	298.0 ± 17.3	1.9 ± 1.6
	[400, 500)	38.3 ± 3.3	8.1 ± 1.5	0.2 ± <0.1	46.6 ± 3.7	47.6 ± 3.1	47.0 ± 6.9	0.6 ± 0.4
	[500, $\infty$)	6.0 ± 1.0	3.7 ± 0.7	0.1 ± <0.1	9.9 ± 1.2	11.3 ± 1.1	17.0 ± 4.1	0.2 ± <0.1
${{\textrm{t}} {}{\overline{{{{\textrm{t}}}}}}} {\textrm{H}}$ 2Boosted1b	[200, 300)	20.2 ± 3.6	3.8 ± 0.4	0.3 ± 0.3	24.3 ± 3.6	20.4 ± 2.6	14.0 ± 3.7	0.5 ± 0.3
	[300, $\infty$)	6.3 ± 1.4	6.1 ± 0.9	0.1 ± <0.1	12.5 ± 1.7	12.9 ± 1.6	15.0 ± 3.9	0.5 ± 0.4
${{\textrm{t}} {}{\overline{{{{\textrm{t}}}}}}} {\textrm{H}}$ 2Boosted2b	[200, 300)	15.8 ± 2.9	3.9 ± 0.9	0.3 ± <0.1	20.0 ± 3.1	18.0 ± 2.4	15.0 ± 3.9	0.4 ± 0.3
	[300, $\infty$)	5.4 ± 1.3	3.8 ± 0.5	0.1 ± <0.1	9.3 ± 1.4	8.6 ± 1.1	6.0 ± 2.4	0.5 ± 0.4
${{\textrm{t}} {}{\overline{{{{\textrm{t}}}}}}} {\textrm{H}}$ 5j1b	[200, 300)	5279.7 ± 114.4	1703.7 ± 82.8	99.1 ± 78.5	7082.4 ± 161.6	7122.6 ± 127.6	7207.0 ± 84.9	14.4 ± 7.7
	[300, 400)	1135.0 ± 31.8	836.4 ± 50.0	22.5 ± 17.3	1994.0 ± 61.7	1960.9 ± 43.2	1907.0 ± 43.7	7.4 ± 3.8
	[400, 500)	182.2 ± 9.0	267.5 ± 24.9	4.0 ± 2.8	453.6 ± 26.6	438.8 ± 16.2	427.0 ± 20.7	2.7 ± 1.4
	[500, $\infty$)	54.2 ± 3.7	146.0 ± 20.3	1.5 ± 1.0	201.7 ± 20.6	226.2 ± 11.5	221.0 ± 14.9	1.5 ± 0.8
${{\textrm{t}} {}{\overline{{{{\textrm{t}}}}}}} {\textrm{H}}$ 5j2b	[200, 300)	1317.8 ± 47.3	350.0 ± 16.6	11.8 ± 8.5	1679.6 ± 50.9	1635.4 ± 33.9	1602.0 ± 40.0	6.3 ± 4.2
	[300, 400)	188.7 ± 9.2	174.1 ± 10.4	2.7 ± 2.0	365.5 ± 14.1	363.3 ± 10.7	367.0 ± 19.2	2.9 ± 1.8
	[400, 500)	33.6 ± 3.5	53.8 ± 5.1	0.5 ± 0.3	87.9 ± 6.2	86.3 ± 4.5	91.0 ± 9.5	0.9 ± 0.5
	[500, $\infty$)	8.2 ± 1.4	24.6 ± 3.5	0.2 ± <0.1	33.0 ± 3.8	36.8 ± 2.5	36.0 ± 6.0	0.5 ± 0.3
${{\textrm{t}} {}{\overline{{{{\textrm{t}}}}}}} {\textrm{H}}$ 6j1b	[200, 300)	3851.5 ± 87.9	805.5 ± 38.8	85.9 ± 66.3	4742.9 ± 116.7	4672.6 ± 87.1	4632.0 ± 68.1	12.3 ± 8.1
	[300, 400)	876.0 ± 27.5	438.8 ± 26.1	19.5 ± 13.4	1334.2 ± 40.2	1332.5 ± 30.4	1371.0 ± 37.0	6.7 ± 4.0
	[400, 500)	179.6 ± 8.5	162.8 ± 15.4	3.4 ± 2.5	345.9 ± 17.8	330.9 ± 11.4	312.0 ± 17.7	2.4 ± 1.4
	[500, $\infty$)	61.0 ± 4.0	98.2 ± 13.6	1.3 ± 1.0	160.5 ± 14.3	179.1 ± 8.4	197.0 ± 14.0	1.6 ± 0.8
${{\textrm{t}} {}{\overline{{{{\textrm{t}}}}}}} {\textrm{H}}$ 6j2b	[200, 300)	1214.0 ± 38.7	237.2 ± 11.4	15.6 ± 12.0	1466.8 ± 42.1	1433.1 ± 29.9	1404.0 ± 37.5	7.8 ± 6.1
	[300, 400)	237.9 ± 12.0	118.8 ± 7.1	3.6 ± 2.9	360.3 ± 14.2	351.9 ± 10.8	341.0 ± 18.5	3.8 ± 2.9
	[400, 500)	38.8 ± 3.8	40.9 ± 4.0	0.6 ± 0.4	80.3 ± 5.6	79.9 ± 4.3	91.0 ± 9.5	1.4 ± 1.0
	[500, $\infty$)	12.9 ± 1.7	21.6 ± 3.0	0.2 ± <0.1	34.7 ± 3.5	38.1 ± 2.4	41.0 ± 6.4	0.7 ± 0.4
VH 2j0b	[200, 300)	17753.9 ± 373.6	29102.3 ± 655.5	105.8 ± 68.3	46962.1 ± 757.6	47499.1 ± 460.7	47559.0 ± 218.1	185.6 ± 92.5
	[300, 400)	2535.2 ± 69.4	5505.3 ± 155.0	16.8 ± 12.0	8057.3 ± 170.3	8075.7 ± 106.8	8106.0 ± 90.0	44.3 ± 23.0
	[400, 500)	278.9 ± 16.1	684.1 ± 34.7	2.8 ± 1.8	965.8 ± 38.3	944.5 ± 26.7	938.0 ± 30.6	6.6 ± 3.4
	[500, $\infty$)	19.2 ± 3.1	76.9 ± 8.1	0.9 ± 0.5	97.1 ± 8.7	95.7 ± 6.6	98.0 ± 9.9	0.6 ± 0.3
VH 2j1b	[200, 300)	3020.1 ± 84.0	2490.4 ± 114.7	26.2 ± 24.5	5536.8 ± 144.3	5808.6 ± 111.1	5883.0 ± 76.7	20.3 ± 10.0
	[300, 400)	360.1 ± 17.3	609.0 ± 44.1	3.6 ± 3.0	972.7 ± 47.5	962.3 ± 30.1	949.0 ± 30.8	5.2 ± 2.8
	[400, $\infty$)	36.3 ± 4.5	66.7 ± 7.3	0.6 ± 0.5	103.7 ± 8.6	111.3 ± 7.7	120.0 ± 11.0	0.7 ± 0.4
VH 2j2b	[200, 300)	209.4 ± 14.0	422.3 ± 46.6	2.0 ± 1.2	633.7 ± 48.6	620.1 ± 26.8	617.0 ± 24.8	10.8 ± 7.9
	[300, $\infty$)	30.7 ± 3.5	102.6 ± 15.4	0.2 ± <0.1	133.6 ± 15.8	131.1 ± 9.8	128.0 ± 11.3	3.5 ± 2.5

**Fig. 6:**
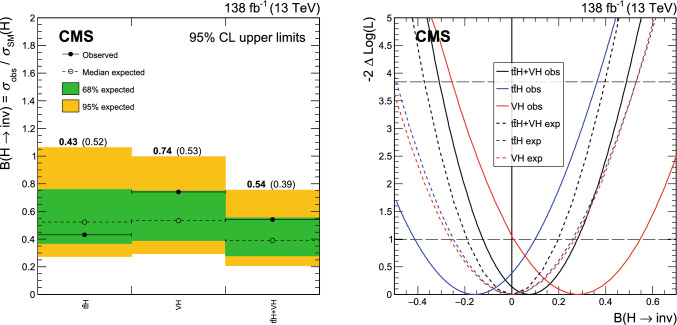
Left: observed and expected limits at 95% $\text {CL}$ for the ${{\textrm{t}} {}{\overline{{{{\textrm{t}}}}}}} {\textrm{H}}$ and VH categories using 2016–2018 data. Right: the profile likelihood scan corresponding to observed and expected (where ${\mathcal {B}({\textrm{H}} \rightarrow \text {inv})} =0$) limits in the fit to the ${{\textrm{t}} {}{\overline{{{{\textrm{t}}}}}}} {\textrm{H}}$ and VH categories

The best-fit value for $\hat{\mu }$ and corresponding 68 and 95% $\text {CL}$ confidence intervals are extracted following the procedure outlined in Refs. [87, 88]. The computing of upper limits adheres to the CLs criterion [89, 90] under the asymptotic approximation [83]. The upper limits on ${\mathcal {B}({\textrm{H}} \rightarrow \text {inv})}$ as extracted from the likelihood fit presented in Sect. 7.1 are found to be 0.43 (0.52 expected) and 0.74 (0.53 expected) at 95% $\text {CL}$ for the ${{\textrm{t}} {}{\overline{{{{\textrm{t}}}}}}} {\textrm{H}}$ and VH categories, respectively, with a combined upper limit of 0.54 (0.39 expected). These results are shown in Fig. 6 together with the observed and expected profile likelihood distribution. The expected distribution assumes ${\mathcal {B}({\textrm{H}} \rightarrow \text {inv})} =0$. The results are compatible with the background expectation. The best-fit ${\mathcal {B}({\textrm{H}} \rightarrow \text {inv})}$ for the ${{\textrm{t}} {}{\overline{{{{\textrm{t}}}}}}} {\textrm{H}}$ and VH categories is $\hat{\mu }=0.07_{-0.10}^{+0.10}(\text {stat.})$ $_{-0.17}^{+0.18}(\text {syst.})$ ($0.00_{-0.10}^{+0.10}(\text {stat.})$ $_{-0.16}^{+0.17}(\text {syst.})$ expected), where the pre-fit normalisation assumes that ${\mathcal {B}({\textrm{H}} \rightarrow \text {inv})}$ $=1$. The systematic uncertainty with the largest impact on the ${\mathcal {B}({\textrm{H}} \rightarrow \text {inv})}$ measurement for the ${{\textrm{t}} {}{\overline{{{{\textrm{t}}}}}}} {\textrm{H}}$ and VH categories using 2016–2018 data are those associated with the JES, while the statistical uncertainty contributes significantly to the overall uncertainty on ${\mathcal {B}({\textrm{H}} \rightarrow \text {inv})}$. The breakdown of the impacts into uncertainty groups are presented in Table 7, together with the expectation assuming ${\mathcal {B}({\textrm{H}} \rightarrow \text {inv})}$ $=0$. The best-fit estimate for the ${{\textrm{t}} {}{\overline{{{{\textrm{t}}}}}}} {\textrm{H}}$ (VH) category is $\hat{\mu }=-0.16_{-0.26}^{+0.26}$ $(0.00_{-0.25}^{+0.26})$ ($\hat{\mu }=0.28_{-0.27}^{+0.27}$ $(0.00_{-0.26}^{+0.27})$).

## Combined ${\textrm{H}} \rightarrow \text {inv}$ limits

A variety of production modes of the Higgs boson can be used for searches for ${\textrm{H}} \rightarrow \text {inv}$ decays. A combination of the results of this analysis, analyses covering the years 2016–2018, and earlier published CMS combination results using Run 1 (years 2011–2012) and 2015 data [30] at $\sqrt{s}=7,~8,~$and 13$\,\text {Te}\hspace{-.08em}\text {V}$, detailed in Table 8, is performed by means of a combined likelihood fit in which systematic uncertainties are correlated across search regions where appropriate. Unless explicitly specified below, parameters of the individual likelihood functions are treated as independent.

**Table 7:** The observed and expected impacts on ${\mathcal {B}({\textrm{H}} \rightarrow \text {inv})}$ for different groups of uncertainties, where the expected results are produced with ${\mathcal {B}({\textrm{H}} \rightarrow \text {inv})}$ $=0$

Uncertainty group	Impact on ${\mathcal {B}({\textrm{H}} \rightarrow \text {inv})}$	
	Observed	Expected
Jet energy calibration	$\pm 0.11$	$\pm 0.11$
Lepton veto	$\pm 0.05$	$^{+0.05}_{-0.04}$
Lepton/photon identification	$\pm 0.06$	$\pm 0.06$
Theory	$^{+0.07}_{-0.06}$	$^{+0.06}_{-0.05}$
Integrated luminosity/pileup	$\pm 0.02$	$^{+0.02}_{-0.03}$
QCD prediction	$\pm 0.02$	$\pm 0.02$
Boosted object/b jet tagging	$\pm 0.02$	$\pm 0.02$
Triggers	$\pm 0.04$	$\pm 0.03$
Stat. uncertainty of simulation	$\pm 0.08$	$\pm 0.08$
Stat. uncertainty in data	$\pm 0.10$	$\pm 0.10$

**Table 8:** Data sets and their respective integrated luminosities used for each production mode across Run 1 and Run 2. For some data-taking periods, no ${\textrm{H}} \rightarrow \text {inv}$ search have been performed for the given production mode, and are not included in the combination

Analysis tag	Production mode	Integrated luminosity (fb$^{-1}$)		
		7$\,\text {Te}\hspace{-.08em}\text {V}$	8$\,\text {Te}\hspace{-.08em}\text {V}$	13$\,\text {Te}\hspace{-.08em}\text {V}$ (Run 2)
VBF-tagged	VBF	–	19.2 [91]	140 [30, 36]
VH-tagged	${\textrm{Z}} (\ell \ell ){\textrm{H}}$	4.9 [91]	19.7 [91]	140 [30, 34]
	${{\textrm{Z}} ({\textrm{b}\bar{\textrm{b}}}){\textrm{H}}}$	–	18.9 [91]	–
	${{\textrm{V}} (\text {jj}){\textrm{H}}}$	–	19.7 [92]	140 [30], [this paper]
	Boosted VH	–	–	138 [35]
${{\textrm{t}} {}{\overline{{{{\textrm{t}}}}}}} {\textrm{H}}$-tagged	${{\textrm{t}} {}{\overline{{{{\textrm{t}}}}}}} {\textrm{H}}$ (hadronic)	–	–	138 [this paper]
	${{\textrm{t}} {}{\overline{{{{\textrm{t}}}}}}} {\textrm{H}}$ (leptonic)	–	–	138 [31, 32]
ggH-tagged	ggH	–	19.7 [92]	140 [30, 35]

For the ${{\textrm{t}} {}{\overline{{{{\textrm{t}}}}}}} {\textrm{H}}$ analysis with fully leptonic final states, a reinterpretation of the supersymmetry searches in the semileptonic and dileptonic t $\overline{{{{\textrm{t}}}}}$ decay channels in Refs. [31, 32] in the context of the t $\overline{{{{\textrm{t}}}}}$  $+$ DM model studied in Ref. [33] has been performed. Another leptonic channel included in this combination is from the ${{\textrm{Z}} (\ell \ell ){\textrm{H}}}$ analysis [34] using 2016–2018 data.

Analyses with hadronic final states partially overlap in their phase space selection, and this must be accounted for in the statistical combination. Those affected by overlap are the VBF analysis [36], the analysis targetting hadronic ggH and boosted VH final states [35], and the resolved VH channel described in this paper.

To remove the overlap between the VBF analysis and ggH/boosted VH analysis, events are considered for rejection in the ggH/boosted VH analysis if they have at least two AK4 jets each with $|\eta | < 4.7$. Specifically, an inversion of the VBF kinematic selection is applied similarly to the ${{\textrm{t}} {}{\overline{{{{\textrm{t}}}}}}} {\textrm{H}}$ and resolved VH analysis as described in Sect. 5.2. These requirements mirror the selection used to enhance the characteristic VBF phase space in Ref. [36], with negligible effect on the sensitivity of the ggH/boosted VH analysis to ${\mathcal {B}({\textrm{H}} \rightarrow \text {inv})}$.

The overlap between the ggH/boosted VH analysis and the VH 2j0b category of this analysis is driven by the low-purity VH category of the boosted analysis. By removing events from the low-purity boosted VH category that contain exactly two AK4 jets forming a dijet candidate with ${65<m_{\text {jj}} <120\,\text {Ge}\hspace{-.08em}\text {V}}$, there is negligible reduction in the exclusion sensitivity of that analysis. The overlap meanwhile is reduced from 30-40% in the CR phase spaces to about 1%.

The uncertainties in the overall cross section for the signal processes are treated as correlated amongst analysis channels, and amongst data sets with the same centre-of-mass energy. The uncertainties related to missing higher-order corrections, as well as PDF variations, are obtained from Ref. [60]. In some of the channels, additional uncertainty contributions relating to signal acceptance modelling are considered. These are treated as uncorrelated amongst the different analysis channels.

The main sources of theoretical modelling uncertainties in the background estimate vary for the different analysis channels. The analyses preferentially select different phase space regions, and employ different assumptions for the modelling of theoretical uncertainties in transfer factors amongst different analysis regions. The resulting uncertainties are therefore treated as uncorrelated.

Significant correlations appear in the treatment of experimental uncertainties. The determination of the integrated luminosity estimate is affected by a number of sources of uncertainty, which are assumed to be correlated amongst all channels, and partially correlated amongst data sets. Some of the analysis channels share trigger requirements, and the uncertainties in the efficiencies of these common triggers are assumed to be correlated amongst channels and uncorrelated amongst data sets. Furthermore, analysis channels often share criteria used for identifying b-tagged jets, as well as the hadronic decay products of tau leptons. The uncertainties in the efficiencies of these identification criteria are assumed to be correlated amongst channels using the same criteria in the same data set. Finally, uncertainties in the calibration of the JER and JES are treated as correlated amongst this analysis, the VBF, and the ggH/boosted VH channels. All other experimental uncertainties are assumed to be uncorrelated amongst channels. For earlier analyses using Run 1 and 2015 data, the correlation scheme established in Ref. [30] is used.

**Fig. 7:**
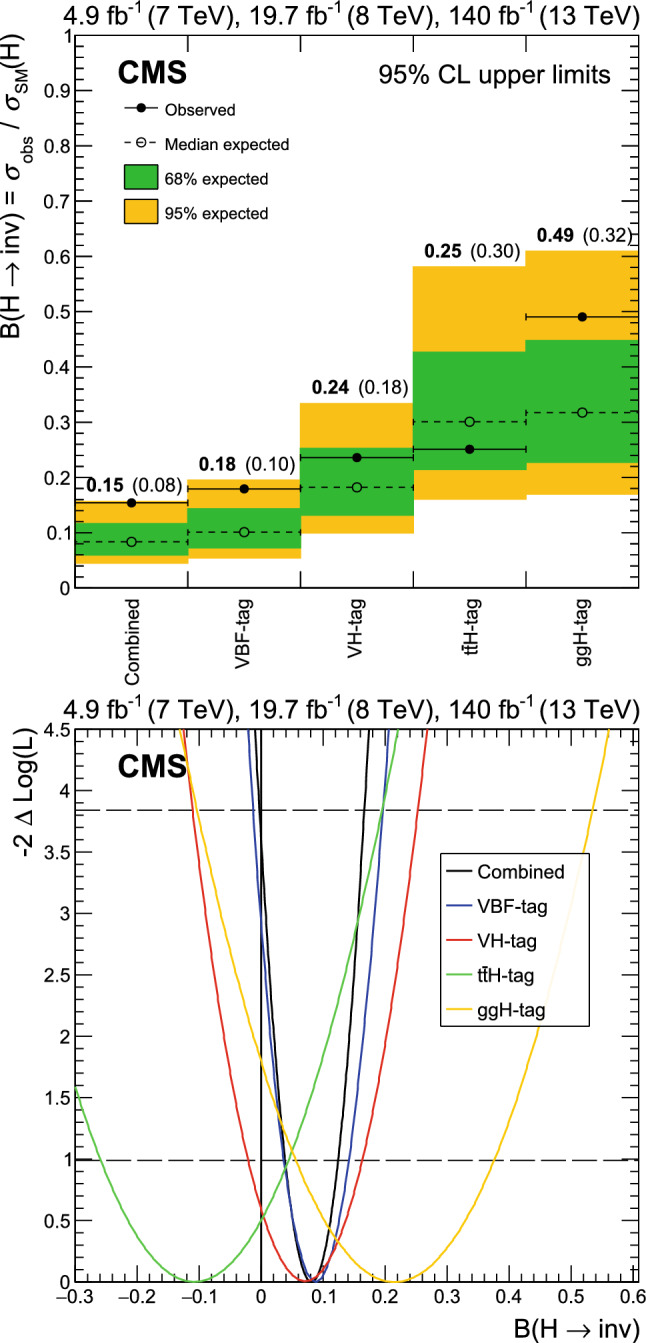
Top: exclusion limits at 95% $\text {CL}$ on ${\mathcal {B}({\textrm{H}} \rightarrow \text {inv})}$. The results are shown separately for each Higgs boson production mode as tagged by the input analyses for Run 1 and Run 2, as well as combined across modes. Bottom: scan of the profile negative log-likelihood as a function of ${\mathcal {B}({\textrm{H}} \rightarrow \text {inv})}$ broken down by the Higgs boson production mode as tagged by the input analyses for Run 1 and Run 2

Exclusion limits on ${\mathcal {B}({\textrm{H}} \rightarrow \text {inv})}$ are calculated assuming SM production cross sections. The 2016–2018 data yields an overall limit of 0.16 (0.09 expected). If the Run 1 and 2015 data-taking periods are included, values larger than 0.15 (0.08 expected) are excluded at 95% $\text {CL}$. This value is dominated by the VBF channel, which yields a limit for ${\mathcal {B}({\textrm{H}} \rightarrow \text {inv})}$ of 0.18 (0.10 expected). The limits for Run 1 and Run 2, separated by the Higgs boson production mode as tagged by the input analyses, are presented in Fig. 7. The integrated luminosities of the Run 1 and Run 2 data sets [30, 33–36] are described in Table 8. The final combination represents an improvement in sensitivity of approximately 20% relative to the most sensitive single channel (VBF).

Maximum likelihood fits to the individual production channels are performed, as well as to the combination of all channels. The dependence of the profile negative log-likelihood functions on the signal strength parameter $\hat{\mu }$ is shown in Fig. 7 (right). The best-fit values of $\hat{\mu }$ for the individual production channels are compatible with one another and with the combined value of ${0.08^{+0.04}_{-0.04}}$, and the observed signal strength is compatible with the absence of a ${\textrm{H}} \rightarrow \text {inv}$ signal within two standard deviations. A breakdown of the best-fit values of $\hat{\mu }$ for each channel are presented in Table 9. A saturated goodness-of-fit test is performed using the final combined likelihood function [93], yielding a probability of 12% that the S$+$B model is consistent with the observed results from the CMS experiment. Tabulated yields and fit results are provided in HEPData [94].

**Table 9:** The observed best-fit estimates of ${\mathcal {B}({\textrm{H}} \rightarrow \text {inv})}$, for each analysis channel in the combination, and the 95% $\text {CL}$ observed and expected (exp) upper limits on ${\mathcal {B}({\textrm{H}} \rightarrow \text {inv})}$

Channel	Best-fit ${\mathcal {B}({\textrm{H}} \rightarrow \text {inv})}$	${\mathcal {B}({\textrm{H}} \rightarrow \text {inv})}$
Combined	$0.08^{+0.04}_{-0.04}$	0.15 (0.08 exp)
VBF-tag	$0.09^{+0.05}_{-0.05}$	0.18 (0.10 exp)
VH-tag	$0.07^{+0.09}_{-0.09}$	0.24 (0.18 exp)
${{\textrm{t}} {}{\overline{{{{\textrm{t}}}}}}} {\textrm{H}}$-tag	$-0.11^{+0.15}_{-0.15}$	0.25 (0.30 exp)
ggH-tag	$0.22^{+0.16}_{-0.16}$	0.49 (0.32 exp)

**Fig. 8:**
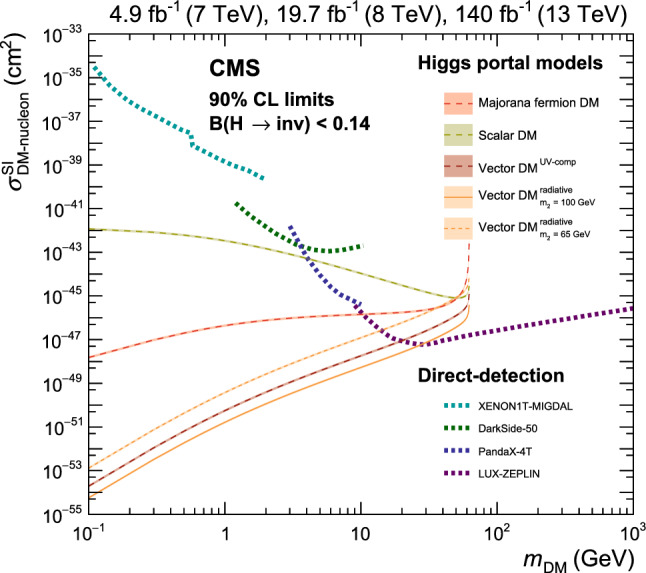
Upper limits on $\sigma ^{\text {SI}}_{\text {{DM{-}nucleon}}}$ as a function of DM candidate mass $m_{\text {DM}}$. Results are presented for a fermion (red) and scalar (yellow) DM candidate. In addition, a vector DM candidate is studied using two UV-complete approaches, the first denoted Vector DM$^{\text {UV-complete}}$ [20] (burgundy), and the second a radiative portal version denoted Vector DM$^{\text {radiative}}_{m_{2}}$ [23] (orange) with a dark Higgs boson mass of $m_2 = 65$ and $100\,\text {Ge}\hspace{-.08em}\text {V}$. Uncertainties are derived from Refs. [19, 100, 101]. Results are compared to direct-detection searches from XENON1T-Migdal [96], DarkSide-50 [97], PandaX-4T [98], and LUX-ZEPLIN [99]

The upper limit on ${\mathcal {B}({\textrm{H}} \rightarrow \text {inv})}$ is interpreted in the context of a set of Higgs portal models of DM interactions, where a stable weakly interacting massive particle (WIMP), such as a singlet scalar, fermion, or vector, has a substantial coupling to a Higgs boson of mass 125$\,\text {Ge}\hspace{-.08em}\text {V}$  [19, 20]. The interaction of a WIMP with an atomic nucleus can occur via the exchange of a Higgs boson, and the resulting nuclear recoil is measured to obtain an upper bound on the spin-independent DM-nucleon scattering cross section, $\sigma ^{\text {SI}}_{\text {{DM{-}nucleon}}}$. An effective field theory (EFT) approach is considered for scalar and fermionic WIMPs, while in the vectorial case two UV-complete DM models are considered, given the EFT appraoch violates unitarity [23, 95]. The vector-spin WIMP model (Vector DM$^{\text {UV-comp}}$) described in Ref. [20], and its radiative portal analogue (Vector DM$^{\text {radiative}}_{m_{2}}$) introduced in Ref. [23] for dark Higgs boson masses $m_2 = 65$ and $100\,\text {Ge}\hspace{-.08em}\text {V}$, and with a mixing angle between the SM and dark Higgs bosons $\theta = 0.2$, are presented. The results are compared to direct-detection searches, where in these experiments it is assumed DM particles interact with atomic nuclei. Direct-detection limits are reported by the XENON1T-Migdal [96], DarkSide-50 [97], Panda-X 4T [98], and LUX-ZEPLIN [99] experiments. Upper limits on $\sigma ^{\text {SI}}_{\text {{DM{-}nucleon}}}$ for DM masses ranging from $0.1\,\text {Ge}\hspace{-.08em}\text {V}$ to $m_{{\textrm{H}}}/2$ are presented in Fig. 8 at the 90% $\text {CL}$ using the full CMS data set. The uncertainties in $\sigma ^{\text {SI}}_{\text {{DM{-}nucleon}}}$ are obtained from the extrema of a coupling parametrisation factor as derived from lattice theory [19, 100, 101]. Results of the Higgs portal interpretation and direct-detection comparison are also provided in HEPData [94].

**Fig. 9:**
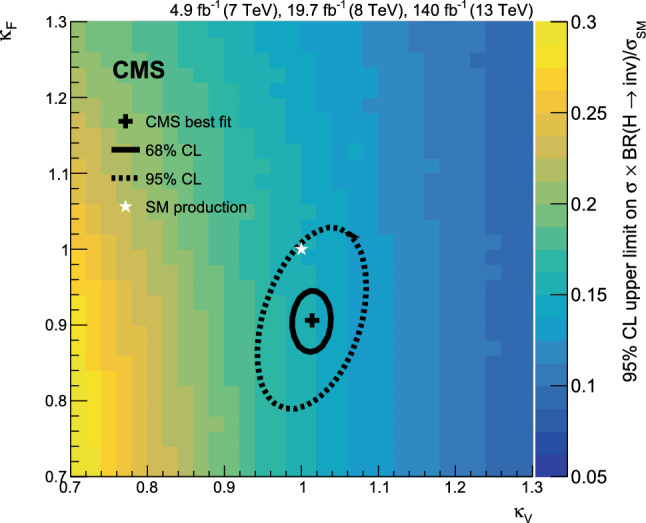
Observed 95% $\text {CL}$ upper limit on ${\mathcal {B}({\textrm{H}} \rightarrow \text {inv})}$ as a function of coupling strength modifiers, $\kappa _{\text {V}}$ and $\kappa _{\text {F}}$, for a Higgs boson of mass 125$\,\text {Ge}\hspace{-.08em}\text {V}$. Best estimates for $\kappa _{\text {V}}$ and $\kappa _{\text {F}}$ from Ref. [11] are shown as a black cross, together with 68 and 95% $\text {CL}$ contours

The sensitivity of the Run 1 and Run 2 combination depends on the cross sections assumed for the different Higgs boson production modes: VBF, VH, ggH, and ${{\textrm{t}} {}{\overline{{{{\textrm{t}}}}}}} {\textrm{H}}$. Cross sections can be parameterised by the coupling strength of the Higgs boson to V bosons and fermions. The cross sections can be directly scaled by coupling strength modifiers $\kappa _{\text {V}}$ and $\kappa _{\text {F}}$ to investigate BSM scenarios [102]. In this context, the observed 95% $\text {CL}$ upper limits on ${\mathcal {B}({\textrm{H}} \rightarrow \text {inv})}$ are evaluated as a function of $\kappa _{\text {V}}$ and $\kappa _{\text {F}}$ and shown in Fig. 9. Best estimates of $\kappa _{\text {V}}$ and $\kappa _{\text {F}}$ from CMS [11] are presented with the 68 and 95% $\text {CL}$ contours. For the best estimate of $\kappa _{\text {V}}$ and $\kappa _{\text {F}}$ by CMS, the 95% $\text {CL}$ limit on ${\mathcal {B}({\textrm{H}} \rightarrow \text {inv})}$ is found to be 0.15 and varies between 0.13 and 0.17 inside the 95% $\text {CL}$ contour.

## Summary

The results of a search for invisible decays of the Higgs boson produced in association with a top-antitop quark pair (${{\textrm{t}} {}{\overline{{{{\textrm{t}}}}}}} {\textrm{H}}$) or a vector boson (VH, where V stands for either a W or Z boson), which decays to a fully hadronic final state, are presented. The analysis is based on proton-proton collision data collected at $\sqrt{s}=13\,\text {Te}\hspace{-.08em}\text {V}$ during the 2016–2018 data-taking period by the CMS experiment at the LHC, corresponding to an integrated luminosity of 138$\,\text {fb}^{-1}$. The ${{\textrm{t}} {}{\overline{{{{\textrm{t}}}}}}} {\textrm{H}}$ production mechanism is investigated using final states containing b jets, or boosted t quarks or W bosons. The VH production channel focuses on resolving a dijet pair with an invariant mass that is compatible with that of a W or Z boson. No significant excess of events is observed above the predicted SM background. A 95% confidence level upper limit of 0.54 (0.39 expected) is set on the branching fraction of the decay of the Higgs boson to an invisible final state, ${\mathcal {B}({\textrm{H}} \rightarrow \text {inv})}$, assuming SM production cross sections.

The results are combined with previous ${\mathcal {B}({\textrm{H}} \rightarrow \text {inv})}$ searches carried out at $\sqrt{s}=7$, 8, and 13$\,\text {Te}\hspace{-.08em}\text {V}$ in complementary production modes. The combined 95% confidence level upper limit on ${\mathcal {B}({\textrm{H}} \rightarrow \text {inv})}$ of 0.15 (0.08 expected) is obtained using Run 1 (2011–2012) and Run 2 (2015–2018) data. The combination represents an improvement in sensitivity of 20% relative to the most sensitive single channel. The results are interpreted in the context of a set of Higgs portal models of dark matter interactions for dark matter masses in the range $0.1\,\text {Ge}\hspace{-.08em}\text {V}$ and $m_{{\textrm{H}}}/2$. Model-dependent exclusion limits are found to complement direct-detection experiments for light mass dark matter candidates.

## Data Availability

This manuscript has no associated data or the data will not be deposited. [Authors’ comment: Release and preservation of data used by the CMS Collaboration as the basis for publications is guided by the CMS policy as stated in https://cms-docdb.cern.ch/cgibin/PublicDocDB/RetrieveFile?docid=6032 &filename=CMSDataPolicyV1.2.pdf &version=2. CMS data preservation, re-use and open access policy.]
